# Public sentiment analysis on urban regeneration: A massive data study based on sentiment knowledge enhanced pre-training and latent Dirichlet allocation

**DOI:** 10.1371/journal.pone.0285175

**Published:** 2023-04-27

**Authors:** Kehao Chen, Guiyu Wei

**Affiliations:** 1 School of Management Science and Real Estate, Chongqing University, Chongqing, China; 2 School of Geography and Ecotourism, Southwest Forestry University, Yunnan, China; University of Sargodha, PAKISTAN

## Abstract

**Background:**

Public satisfaction is the ultimate goal and an important determinant of China’s urban regeneration plan. This study is the first to use massive data to perform sentiment analysis of public comments on China’s urban regeneration.

**Methods:**

Public comments from social media, online forums, and government affairs platforms are analyzed by a combination of Natural Language Processing, Knowledge Enhanced Pre-Training, Word Cloud, and Latent Dirichlet Allocation.

**Results:**

(1) Public sentiment tendency toward China’s urban regeneration was generally positive but spatiotemporal divergences were observed; (2) Temporally, public sentiment was most negative in 2020, but most positive in 2021. It has remained consistently negative in 2022, particularly after February 2022; (3) Spatially, at the provincial level, Guangdong posted the most comments and Tibet, Shanghai, Guizhou, Chongqing, and Hong Kong are provinces with highly positive sentiment. At the national level, the east and south coastal, southwestern, and western China regions are more positive, as opposed to the northeast, central, and northwest regions; (4) Topics related to Shenzhen’s renovations, development of China’s urban regeneration and complaints from residents are validly categorized and become the public’s key focus. Accordingly, governments should address spatiotemporal disparities and concerns of local residents for future development of urban regeneration.

## Introduction

Urban regeneration is currently one of the most pressing issues in global urban research. Urbanization has shifted from the growth of new spaces to the regeneration of existing communities. Urban regeneration projects are effective tools for improving urban competitiveness, improving the quality of urban housing, and narrowing the wealth gap [1]. Several countries have implemented urban regeneration initiatives to make necessary and planned changes to areas of cities that are no longer suitable for modern urban living, including in the United States [2], Ireland [3], and Japan [4] among developed countries, as well as China [5], Turkey [6], and Russia [7] among developing countries.

China’s urban regeneration initiatives entered a period of full-scale advancement with the central government’s formal guidance in July 2020 [8]. According to the "General Office of the State Council on the Comprehensive Promotion of Urban Old Neighborhood Renovation Work Guidance," the renovation of China’s old neighborhoods is a major livelihood and development project necessary to meet the needs of the people for a better life. In this context, public satisfaction is the ultimate goal of China’s urban regeneration plan [9]. In reality, China’s urban regeneration measures have not adequately addressed the concerns of local residents [10]. The recent proliferation of demolition conflicts [11], serious decision challenges [12], and incidents of public-government conflict [13] have resulted from decision makers, implementers, and managers of urban regeneration projects ignoring or weakening public participation and opinion sharing [1]. It is therefore imperative that the public’s true desire and sentimental expressions for urban regeneration be valued and investigated as widely as possible.

Limited research has been conducted on public sentiment analysis in the context of urban regeneration. Several studies have shown that public involvement in the decision-making [14] and implementation [15] phases of urban regeneration projects is the most effective way to reflect public opinion and preferences. In contrast, the process of examining public sentiment for singular projects lacks a global perspective, resulting in a distorted or even misguided vision of macro judgments and evaluations for urban regeneration. Some scholars have conducted semi-qualitative questionnaires [16], opinion interviews [17, 18], literature reviews, and case studies [19] on the attitudes and commentary of key stakeholders toward specific industrial regeneration projects that provide targeted individual-level data; however, the results may be subject to selection and response biases [20] and may not objectively reflect the overall sentiment tendency of society and the priorities of people’s demands for urban regeneration. Currently, no reliable method exists for analyzing public sentiment on urban regeneration in a broad, objective, and precise manner, preventing an accurate assessment of the public’s sentiments and focus on urban regeneration. Due to this gap, governments and practitioners are unable to judge the important social benefits of urban regeneration and whether it meets the related expectations based on people’s long-cherished wish for a better life.

In this context, this study utilizes an advanced deep learning (DL) -based Sentiment Knowledge Enhanced Pre-training (SKEP) [21] model to classify public sentiment on urban regeneration. SKEP helps to improve the accuracy of the sentiment classification tasks. Furthermore, we adopts a word cloud (WC) to analyze public focus on urban regeneration and an advanced traditional machine learning (ML) model–Latent Dirichlet Allocation (LDA) [22]–to categorize themes of public comments. The key research contributions and innovations of this study are summarized as follows:

- In spite of the fact that social media (Weibo) has been proven to be a reliable tool for capturing public sentiment [23, 24], public feedback and questions from forums and government platforms have not been sufficiently considered. In order to address this limitation, this study uses data from Weibo, Chinese forum websites, and Chinese government affairs platforms to classify public sentiments on urban regeneration.
- Three of the most advanced DL-based sentiment classification models, BERT, RoBERTa, and SKEP, are adopted and tested. By comparing several widely adopted performance metrics, SKEP is employed to achieve state-of-the-art accuracy in sentiment classification tasks, maintaining the superior robustness and applicability of the results.
- Divergences of public sentiment tendency on China’s urban regeneration are observed and analyzed, including spatial and temporal distributive characteristics, public focus and comment topic categorization, which enriches limited surveys in gauging temporal dynamics and geographical variations in the public sentiment analysis domain [25].
- By identifying and visualizing the focus of public commentary on urban regeneration based on WC and categorizing themes of public comments based on LDA, this study provides a clear understanding of the context of public commentary on urban regeneration.

The results suggest that the public sentiment was generally positive on China’s urban regeneration, but varied widely over time and space. Public comments were categorized into three topics concentrating on Shenzhen’s innovations, strategic planning of urban regeneration initiatives and complaints by residents. The findings of this study have policy implications and are relevant to addressing spatiotemporal disparities and residents’ concerns regarding Chinese urban regeneration initiatives in the future. We also discussed the current developments and approaches in sentiment analysis domain and compared SKEP with the existing DL-based benchmark models to demonstrate its state-of-the-art performance.

## Literature review and background

### Public participation in urban regeneration

Research on public participation in urban regeneration has generated considerable interest, particularly regarding the influence of residents’ comments during the decision-making and implementation phases of the urban regeneration process.

In the decision-making phase of regeneration projects, Sager’s study on social participation in community regeneration planning indicated that planning advocacy is generally successful [26]. Based on a two-stage model, Wang developed a group decision approach to solve the problem of traditional regeneration projects’ decision making [1]. Liu examined how the proactive behavior of government officials influences citizens’ satisfaction with urban regeneration policies through the policy implementation process theory [27]. Williamson examined how contestation and resistance can influence government-led urban regeneration initiatives [13]. By incorporating in-depth interviews from a government perspective, Cao demonstrated how public participation can be used to restore residents’ rights in urban regeneration projects [12].

In the implementation phase of regeneration projects, based on the meme theory, Zhao et al. constructed a neighborhood micro-regeneration system and demonstrated three scenarios of public participation from the perspective of micro-participants and micro-objects [28]. Liu et al. found that the degree and normative nature of public participation may be key factors contributing to the cooperative behavior of urban regeneration stakeholders [15]. A study conducted by Hui et al. examined the role of public participation in community planning and how communities can regain vitality and public favor by practicing community regeneration [29]. By surveying government administrators and the general public in China, Xiao analyzed the key factors of public participation effectiveness in the urban regeneration process [30].

Observations from the above literature indicate that research on public participation in urban regeneration is primarily conducted during the decision-making and implementation phases. Among the issues explored are residents’ participation in regeneration projects, the decision-making mechanism of regeneration projects, and the influence and importance of residents’ comments on the initiation and operation of regeneration projects.

The nature of urban regeneration projects, however, differs from that of general construction projects in that they are oriented toward the needs of people; focusing solely on public commentary and participation during the decision-making and implementation phases cannot provide in-depth information on the outcomes of urban regeneration projects, nor can they comprehensively evaluate the performance of regeneration projects and guide the government in implementing future regeneration projects. As such, the overall public commentary on urban regeneration requires extensive examination over a prolonged period, which is one of the primary objectives of this study.

### Public commentary on urban regeneration

Public commentary regarding urban regeneration has not been studied extensively. Using social network analysis methods, Cao and Tang compared networks of public space layouts and residents’ daily behaviors in urban regeneration, and evaluated the effects of the regeneration process [31]. A study conducted by Darnthamrongkul and Mozingo examined the responses of users regarding 16 urban stormwater management projects located in the San Francisco Bay Area, thereby providing empirical insights into how citizens are implementing low-impact urban regeneration practices [32]. Based on qualitative research and a narrative survey methodology, Jelili et al. evaluated an urban regeneration program implemented in Lagos Island and found that resident input should be encouraged in order to achieve effective resident cooperation [33].

A few scholars have focused on public comment analysis and the evaluation of old industrial renovation projects, which is one of the most important components of urban regeneration. Using focus group interviews and literature, as well as secondary sources, Kim examined the reasons why old industrial renovation did not occur in Seoul [34]. Huang conducted an evaluation of the performance of old industrial buildings after regeneration using a public questionnaire and the structural equation model and importance–performance analysis model [16]. The results of He et al.’s study on the expectations of stakeholders in China’s old industrial renovation projects revealed significant differences in the economic, social, and environmental expectations of different stakeholder groups [18]. Using the concept of regional industrial ecosystems, Han and Sanghoon examined insider perceptions of local governmental urban regeneration initiatives and offered recommendations for future urban regeneration projects [35]. Through a literature review and case study method, Loures examined the perceptions of the general public and experts regarding industrial regeneration [19].

According to the above literature, public commentary on urban regeneration have not been researched extensively. In terms of research subjects, current studies generally focus on a single region or a specific urban regeneration project. Despite the fact that several scholars have noted the importance of public response and reviewed stakeholders’ opinions on old industrial renovation projects, the current studies are not able to gather and examine public opinion on a national scale and analyze its characteristics from a spatiotemporal perspective, which lacks guidance and general applicability. In terms of research methods, the majority of existing studies rely on qualitative analysis, such as questionnaires, structured interviews, and literature surveys, in which human intervention is common, and the results lack an empirical, clear, and objective quantitative analysis; thus, the essence of the phenomenon is not adequately analyzed statistically. However, as urbanization progresses, an increasing number of outdated residential and industrial areas need to be functionalized in order to meet the needs of the city’s functional enhancement from citizens’ willingness to produce residential or public service sites such as commercial complexes [36], urban green spaces [37], and industrial complexes [38]. Further, it is important to conduct an extensive survey of public commentary on urban regeneration. By doing so, the government and project stakeholders can evaluate urban regeneration projects from the perspective of the public, on the one hand. On the other hand, they are capable of identifying people’s demands and pain points for urban regeneration projects during urban development, in order to implement urban regeneration initiatives more effectively and enhance the functional improvement of urban industrial transformation.

### Sentiment analysis in the construction industry

Social media has transformed long-distance informal communication venues into fast and easy communication methods, allowing potential users to access data swiftly and easily [39]. Substantial unstructured data are generated, which provides valuable knowledge and presents great opportunities for businesses, government agencies, and individuals to create new services. The exploitation of this unstructured data has created a new field, namely sentiment analysis, which has been widely applied in areas such as epidemic communication [40], international relations [41], and education policy [42].

In the construction industry, by analyzing public sentiments and comments, a project’s business action plan can be greatly improved, as can the company’s economic, social, and environmental performance. Research has been conducted on sentiment analysis in the construction industry. Using the social cognitive theory, Wang et al. examined the public acceptance and determinants of two mega construction projects in Wuhan, China, using questionnaires and structural equation modeling [43]. He et al. conducted a feedback survey involving 418 residents in the vicinity of two chemical parks in Dalian in the Bohai Rim region using a face-to-face questionnaire and Spearman correlation analysis [44]. Ge et al. validated their social psychological model using questionnaires and structural equation modeling, demonstrating how benefits, risks, and trust affected public acceptance of the S35 Yongjin highway infrastructure in Yunnan Province, China [45]. According to Jiang, Lin, and Qiang’s project, wherein sentiment analysis was performed using a thesaurus-based approach, half of the messages expressed negative sentiment with regard to the Three Gorges Project, whereas the remainder expressed positive or neutral sentiment [46]. A study conducted by Valentin, Naderpajouh, and Abraham examined the impact of technological systems on public opinion in society using infrastructure projects as an example [47]. As part of an analytical framework for thematic modeling and sentiment analysis of public concerns about the Hong Kong-Zhuhai-Macao Bridge, Zhou, Zhou, and Qian provided comments on the management of public opinion on infrastructure projects [48].

Public sentiment analysis in the construction industry focus primarily on large construction and infrastructure projects, using quantitative methods for stakeholders. However, limited research has targeted urban regeneration initiatives that are currently being vigorously promoted across China. In addition, only a few scholars have examined public sentiments through the analysis of massive datasets. Thus, the current literature has several shortcomings.

First, the public sentiment analysis of stakeholders can reveal user preferences to some extent, but such preferences are specific to the project and do not reflect broader public sentiment tendencies. Second, with the proliferation of the Internet, public opinion regarding projects is becoming more prevalent on social media, and the importance of online sentiment in project decisions and evaluations is becoming increasingly evident, especially for projects such as urban regeneration that are highly related to citizens. However, project managers and academics have paid little attention to this emotional feedback on social media platforms. Third, traditional methods of public sentiment analysis, such as questionnaires, structured interviews, and grammatical analysis, are unable to objectively and comprehensively tap into the opinions of social groups; thus, they cannot provide decision-makers and managers with commentary support and universal criteria for evaluation because of the limitations of data accessibility and extensiveness.

In the context of the above discussion, this study is the first to use massive amounts of data from social media, online forums, and government affairs platforms to analyze public sentiments on China’s urban regeneration process. The core research questions are: 1) In China, what is the level of public sentiment regarding urban regeneration? 2) What are the characteristics of distribution of the public sentiment tendencies over time and space? 3) What are the particular focus and themes of public comments on China’s urban regeneration? By answering these questions, not only this study contributes to the current literature and fill the gaps in the urban regeneration domain, but also it provides realistic social evidence and macro-level references for urban regeneration initiatives in China, in order to assist the government and practitioners in the implementation and evaluation of urban regeneration projects.

## Methodology

Sources of data for this study included social media, online forums, and government affairs platforms. The following reasons led to the selection of data sources. First, social media is becoming an increasingly popular medium for expressing opinions and preferences, and it has been used for sentiment analysis on a variety of social issues, including urban issues [49], climate change [50], and health care [51]. Second, online forums are an efficient and thoughtful way to disseminate information with the rapid development of the Internet [52], as they contain a large number of informative topic comments that can be analyzed for their sentiment [53]. Third, government affairs platforms are also an important channel for the public to express their views and demands to the government [54]. Therefore, to obtain as many public comments as possible, this study used data from social media, online forums, and government affairs platforms. To improve the efficiency and accuracy of data collection and processing, and to avoid errors in manual processing due to massive amounts of data, this study used a combination of Natural Language Processing (NLP), an advanced DL architecture-SKEP, and an advanced traditional ML model-LDA to perform the public sentiment analysis. The next section describes how public comments were collected and how they were pre-processed by NLP. We then review the developments and recent approaches in sentiment analysis domain. Following this, DL-based SKEP and existing sentiment classification models are described, and their performance is compared. Last, we present the WC-based public focus analysis method and LDA-based comment topic categorizing method. Our data collection and analysis approaches complied with the terms and conditions for all sources of the data.

### Data collection and the NLP-based pre-processing of public comments

Data used in this study were from: 1) Weibo [55], one of the most dominant social media applications in China as of 2021 [56]; 2) 466 Chinese forum websites; 3) 42 Chinese government affairs platforms. Names of the original websites with English translations are listed in table in S1 Table. This study employed a hypertext mark-up language-based web crawler to obtain public comments from these sources. For comments search regarding urban regeneration, search terms such as "urban regeneration," "old neighborhood renovation," "old neighborhood regeneration," "neighborhood regeneration," "old regeneration," "industrial renovation," and "old industrial site" were used. To ensure that the collected comments were from the public, posts from government, media, campus, corporate, and website accounts were not included. In total, 41,248 comments regarding urban regeneration were collected between 00:00 on December 01, 2020, and 24:00 on June 01, 2022 (S2 Table). The following features were collected for each comment: posting source (accurate to the province), posting time (accurate to the day), and comment content (plain text in Chinese). Based on NLP, the pre-processing of the comments included 1) de-duplication of the posted content, 2) removal of invalid characters within the content, 3) separation of Chinese text, and 4) deactivation of words. In the first step, 29,504 public comments remained after de-duplicating 41,248 public comments. In the second step, all invalid characters in the comment content were removed using regular expressions. In the third step, the Jieba word segmentation tool [57] was used to segment the Chinese text in the comment content. This tool is an open-source toolkit based on Python and has an accuracy rate of over 85%, making it the best choice for Chinese text segmentation [58]. In the fourth step, the Chinese deactivation word list was used to eliminate and deactivate the words of the comment content to provide analyzable text for sentiment classification, focus analysis and topic categorization.

### DL-based sentiment classification models

Numerous techniques exist to perform various tasks in sentiment analysis, including traditional ML supervised approaches [59], as well as unsupervised approaches. Sentiment lexicon and ML methods are among the most widely used [39]. Nevertheless, sentiment-based lexicon analysis is highly dependent on lexicon accuracy (as demonstrated in specific tasks), and has poor generalizability [60]. ML approaches, however, can automatically build sentiment models based on information obtained from a large corpus of data [60]. Yet, traditional ML fails to perform well in sentiment analysis owing to its reliance on feature engineering, which is set in advance and requires the manual extraction of features for training; this is complicated and time-consuming and cannot easily be adapted for new knowledge extraction tasks in other domains [61].

DL is a branch of ML and a mainstream trend in its development [39]. DL enables computers to perform feature computation automatically, which manifests itself in the field of NLP, by embedding text data into a low-dimensional continuous feature vector. As a result of this approach, a computer can descriptively learn abstract feature expressions that describe the essence of the data without the need for human intervention, and many studies have demonstrated the higher reliability of its analysis results [62]. Considerable progress has been made in DL models that use transformer architecture based on self-attentive mechanisms, such as Bidirectional Encoder Representations from Transformers (BERT), Robustly optimized Bidirectional Encoder Representations from Transformers (RoBERTa) [63] and SKEP [21], which use sentence context and lexical affixes to improve the performance of DL models. In this study, we compare the performance of DL-based BERT, RoBERTa, and SKEP model to select the state-of-the-art model for our binary sentiment classification task.

#### BERT

BERT is a neural network-based technique for pre-training natural language processing that was introduced by Google AI in 2018 [64] and has been largely applied to sentiment analysis [65]. BERT is based on transformer architecture, which contains many transformer neural network modules with complex network structures and fast parallelism via a self-attention mechanism. By improving the disadvantage of slow training of traditional language models and increasing their depth to a very deep level, BERT is able to fully exploit the features of language models and improve their accuracy. Through the use of an attention mechanism, BERT determines the context of a word in relation to the other words in a text sequence. The model generates a representation of the words in the corpus by using both the previous and next contexts.

**Fig 1:**
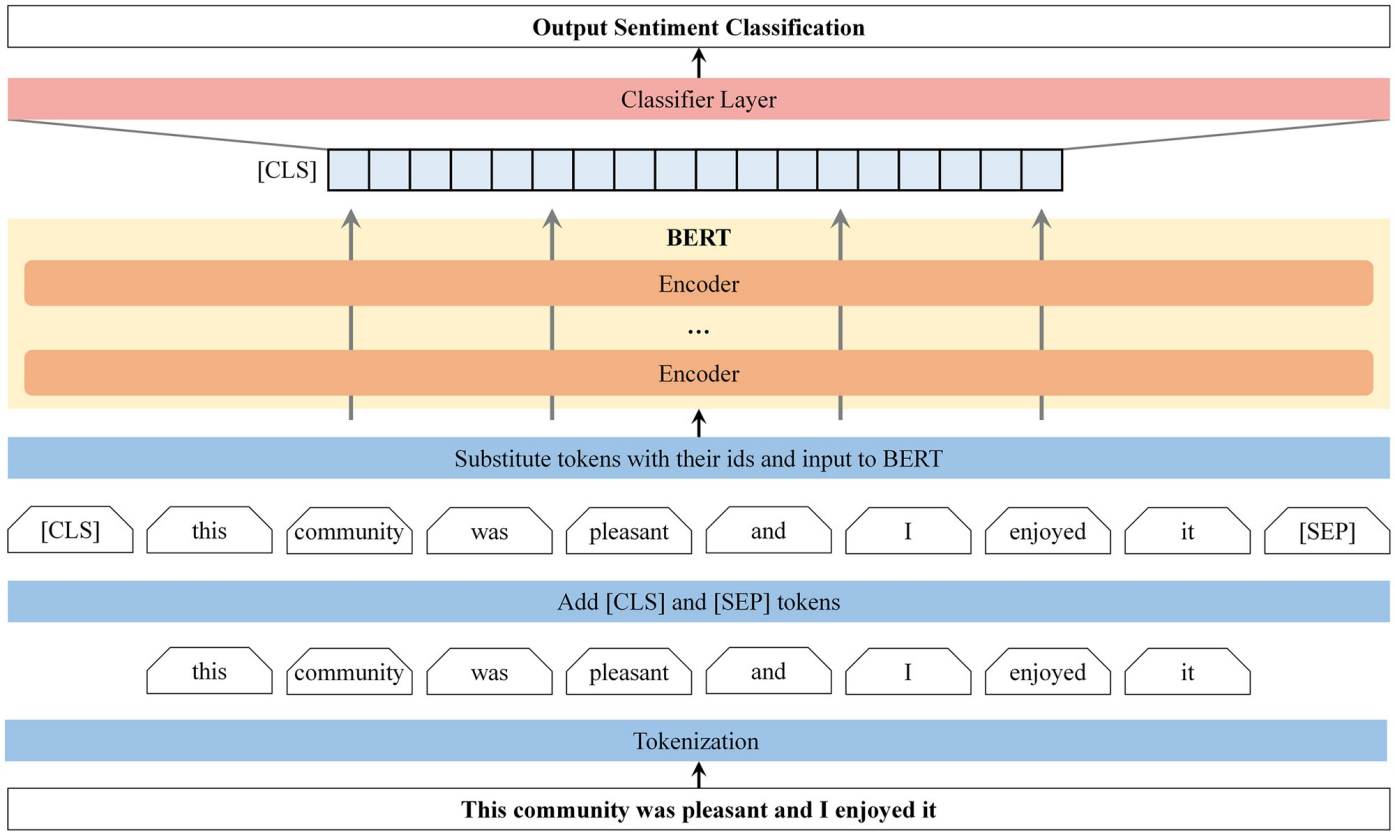
Overview architecture of BERT model. Adapted from Devlin et al. [64].

BERT’s basic structure includes 12 transformer blocks, 768 hidden sizes and 110 million self-attention heads. Fig 1 illustrates the architectural representation of BERT. A BERT model is composed of several encoder layers, as well as a large feed-forward neural network and attention heads [64]. A feed-forward neural network performs self-attention before transferring the results to the next encoder layer. Input to the model consists of a sequence of words beginning with the [CLS] token, while output is a vector representation of the sequence. To represent the input sequence for the classification task, the BERT model uses the final output of the first [CLS] token [66].

In this study, we utilized the standard architecture of BERT, with some modifications to the hyper-parameters that control the learning process in machine learning. We tested multiple parameter combinations on the development set (10 percent of manually annotated comments) in order to optimize the parameters. Upon fine-tuning the parameters, we obtained an effective learning rate of 5×10^−5^ and a total of 768 hidden layers. Additionally, 256 tokens were set as the maximum sequence length, 32 batch sizes were used, and the model was trained for 10 epochs on a GTX 1660 Ti GPU.

#### RoBERTa

RoBERTa model is an improved version of BERT, a deep learning language representation model that uses transformers. The advantages and improvements of RoBERTa over BERT are as follows. First, during pre-training, the next sentence prediction objective is not included. Secondly, RoBERTa employs dynamic masks, which means that each time a sequence is input to the model, a new mask pattern is generated. Through continuous input of large amounts of data, the model gradually adapts to different masking strategies and learns different linguistic representations. Thirdly, RoBERTa is trained on a broader amount of data, with longer sequences, larger batches, and over a longer period of time [67], which allows it to generalize more effectively to sentiment classification tasks than BERT.

The standard RoBERTa architecture was used in our study, with some modifications to the hyper-parameters. Several parameter combinations were tested on the development set (10 percent of manually annotated comments) in order to optimize the parameters. As a result of fine-tuning the parameters, we were able to obtain an effective learning rate of 5×10^−5^ and a total of 768 hidden layers. Moreover, 512 tokens were set as the maximum sequence length, 12 batch sizes were used, and the model was trained for 6 epochs on a GTX 1660 Ti GPU.

#### SKEP

SKEP is a DL architecture for integrating sentiment knowledge through self-supervised training [21]. SKEP integrates different types of sentiment knowledge together to provide a unified sentiment representation for various sentiment analysis tasks. SKEP differs from traditional sentiment analysis methods, which study different types of sentiment knowledge separately for different sentiment tasks. To embed sentiment information into the pre-trained sentiment representation, SKEP used automatically mined knowledge to perform sentiment masking and constructed three sentiment knowledge prediction objectives. In particular, the correlation between aspect and sentiment was captured by transforming pairwise predictions into multi-label classifications.

**Fig 2:**
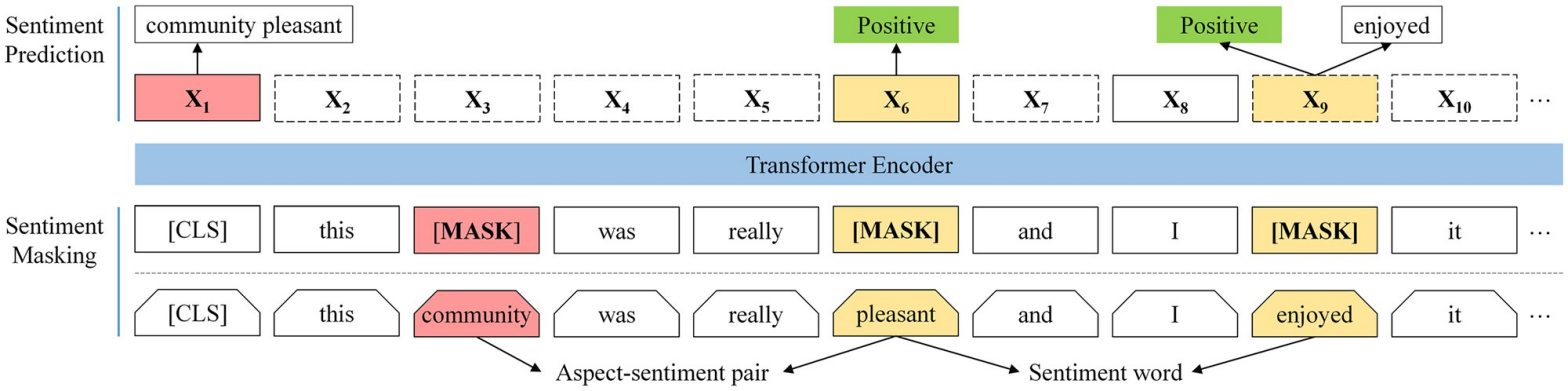
Overview architecture of SKEP model. SKEP model contains two parts: (1) sentiment masking detects sentiment information from an input sequence using automatically mined sentiment knowledge, and generates a corrupted version by removing this information; (2) sentiment pre-training requires the transformer to recover the removed information from the corrupted version. The three prediction objectives on top are jointly optimized: Sentiment word prediction (on X9), word polarity prediction (on X6 and X9), and aspect-sentiment pairs prediction (on X1). Notably, on X6, only word polarity is calculated without the sentiment word, as its original word has been predicted in the aspect-sentiment pairs prediction on X1. Adapted from Tian et al. [21].

Fig 2 illustrates the two main modules of SKEP: sentiment masking and sentiment pre-training.

A major advantage of SKEP is that users do not require a large corpus of text to train their models. It requires only fine-tuning based on region-specific and task-specific supervised data, since it has been pre-trained on a large existing corpus. Moreover, SKEP is conceptually simple, but empirically effective. On most datasets, it outperforms BERT and RoBERTa, which are powerful pre-trained baselines [21].

In this study, we used a PyCharm compiler based on the Python 3.9 environment for model programing. SKEP runtime environment was configured using the Baidu-AIP toolkit [68] in the PyCharm compiler. We then used the open source code of SKEP model architecture [69] to perform binary sentiment classification tasks. SKEP model runs with the output included the sentiment of every comment (positive or negative) and the confidence level of its sentiment classification result (0% to 100%, with higher indicating more valid results for sentiment classification).

#### Performance comparison of the sentiment classification models

We evaluated the sentiment classification performance of BERT, RoBERTa and SKEP model on our 29,504 pre-processed comments in order to select the state-of-the-art model for our binary sentiment classification tasks. The performance of models is compared in Table 1.

**Table 1:** Performance comparison of the sentiment classification models.

Models	Accuracy	Precision	Recall	F1-score
BERT	77.5%	80.3%	87.5%	83.8%
RoBERTa	79.2%	86.8%	65.6%	74.7%
**SKEP**	**81.0%**	**89.3%**	**88.0%**	**85.8%**

According to Table 1, compared to the current state-of-the-art BERT-based RoBERTa [25], SKEP demonstrated the highest accuracy, precision, recall, and F1-score. This outstanding performance is achieved by significant improvements in fine-grained tasks, aspect-level classification, and opinion role labeling as well as new advances in most datasets that are typical of sentiment analysis tasks [21]. Thus, we use SKEP as our sentiment classification model.

### WC-based public focus analysis method

A WC represents the visual analysis results of all comments in units of Chinese words. A WC was statistically sorted and presented according to the frequency of Chinese words in pre-processed comments. In this study, we used the Python 3.9-based PyCharm compiler to create a WC map of all public comments on urban regeneration to visualize the distribution of Chinese words in public comments and public focus. The larger the area occupied by one Chinese word in the WC, the higher the frequency of occurrence, and thus, the higher the public attention it receives.

### LDA-based comment topic categorization method

The LDA topic modeling method, a traditional ML model, relies on clustering to discover potential variables or hidden structures in data. This method significantly reduces the need for human classification intervention and is the highest-performance topic modeling method available [70].

**Fig 3:**
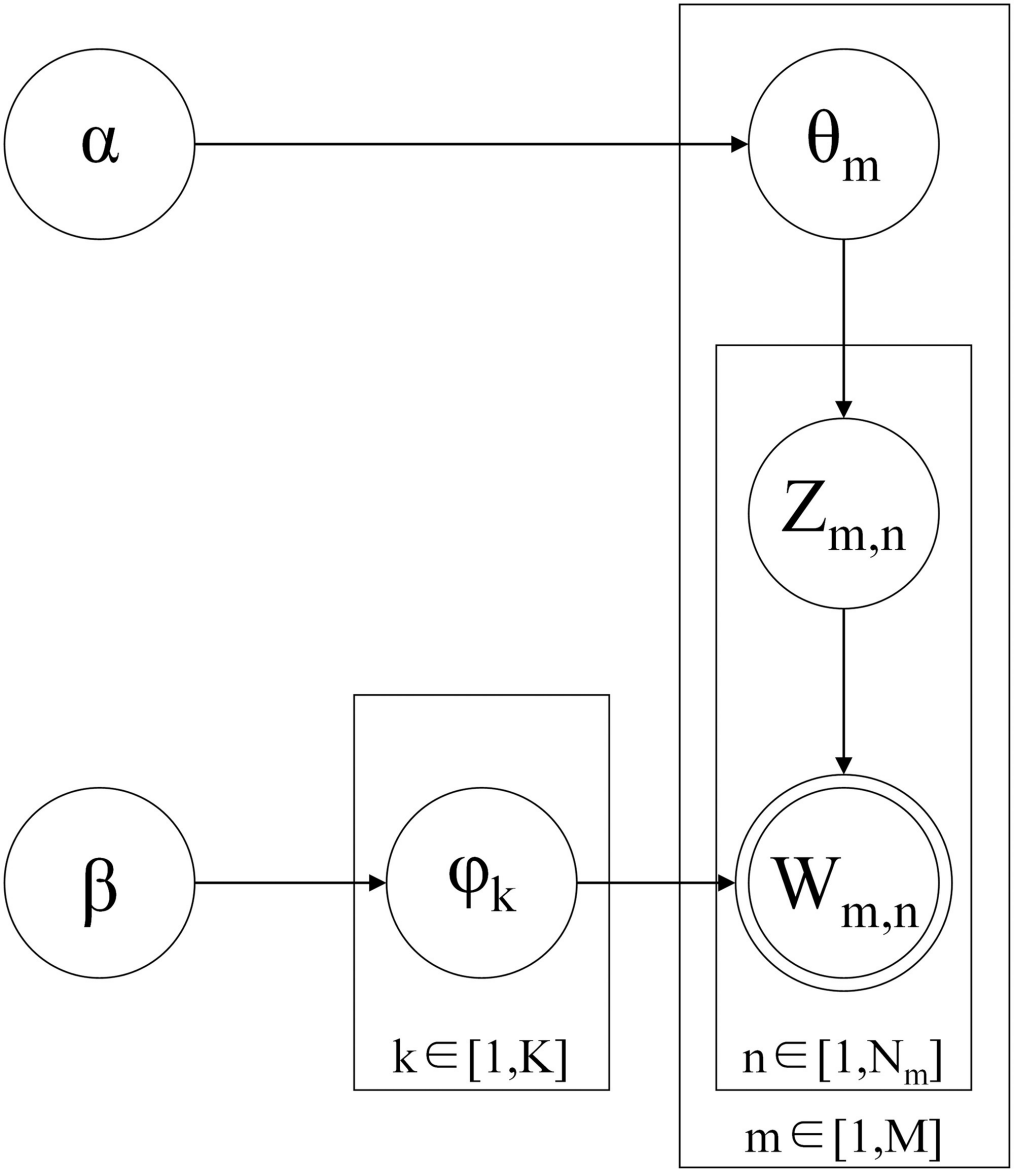
Topic generation process of the LDA model. Adopted from Blei et al. [22].

The topic generation process and the symbolic representation of the LDA model are illustrated in Fig 3. Among them, LDA contains a three-level Bayesian probabilistic model and a topic generation model with observable variables represented as bicircular w and other latent variables represented as unicircular. α is the Dirichlet parameter before each document-topic distribution. β is the Dirichlet parameter before each topic-word distribution. θ_i_ is the topic distribution of document i (sum of θ_i_ is 1.0). φ_k_ is the word distribution of topic k, Z_i,j_ is the topic of the jth word in document i, and W_i,j_ is a specific word. Comment topics are generated as follows: (1) the topic distribution θ_i_ of document i is generated by sampling from the Dirichlet distribution α; (2) the subject Z_i,j_ of the jth word of document i is sampled from the subject polynomial distribution θ_i_; (3) the word distribution φ_k_, (k = Z_i,j_), representing the number of topics corresponding to topic Z_i,j_, is sampled from the Dirichlet distribution β; and (4) the final word W_m,n_ is generated by sampling from φ_k_, which represents the words contained in the topic. Because the optimal number of topics in the LDA model needs to be specified artificially, this study used a combination of topic perplexity [71] and the elbow method [72] to select the optimal number of clustered topics.

The idea of topic perplexity is that there may be an over-clustering problem when the cosine similarity in topic similarity follows a decreasing trend with an increasing number of topics. To address this problem, a perplexity measure must be introduced into the LDA model, which is a standard way to measure the predictive power of the model [73]. The formula for calculating the degree of topic perplexity is as follows:

$$\text{Perplexity}=\text{exp}\left\{-\frac{\sum_{\text{d}=1}^{\text{M}}\;\text{log}\;\text{p}\left({\text{w}}_{\text{d}}\right)}{\sum_{\text{d}=1}^{\text{M}}\;{\text{N}}_{\text{d}}}\right\}$$ (1)

In formula 1, P (w_d_) is the probability of each word appearing in the test set and N_d_ is the total number of all words appearing in the test set. A higher number of topics and lower level of perplexity indicate a model that is more capable of classification. Nevertheless, a large number of topics may lead to overfitting in the LDA model. For this reason, the elbow method was used in this study to avoid overfitting of the LDA model, that is, the optimal number of topics was chosen based on the significant inflection points in the graph.

Having selected the optimal number of topics and completed the topic categorization of the comment content, it is necessary to assess the topic validity based on the inter-topic distance map (IDM). In IDM, the topics are represented by circles of varying sizes; the larger the circle, the greater the number of words contained in the topic. The LDA model is highly valid for categorizing results if no circles cross each other and are far apart.

## Results

### Public sentiment classification on urban regeneration

**Fig 4:**
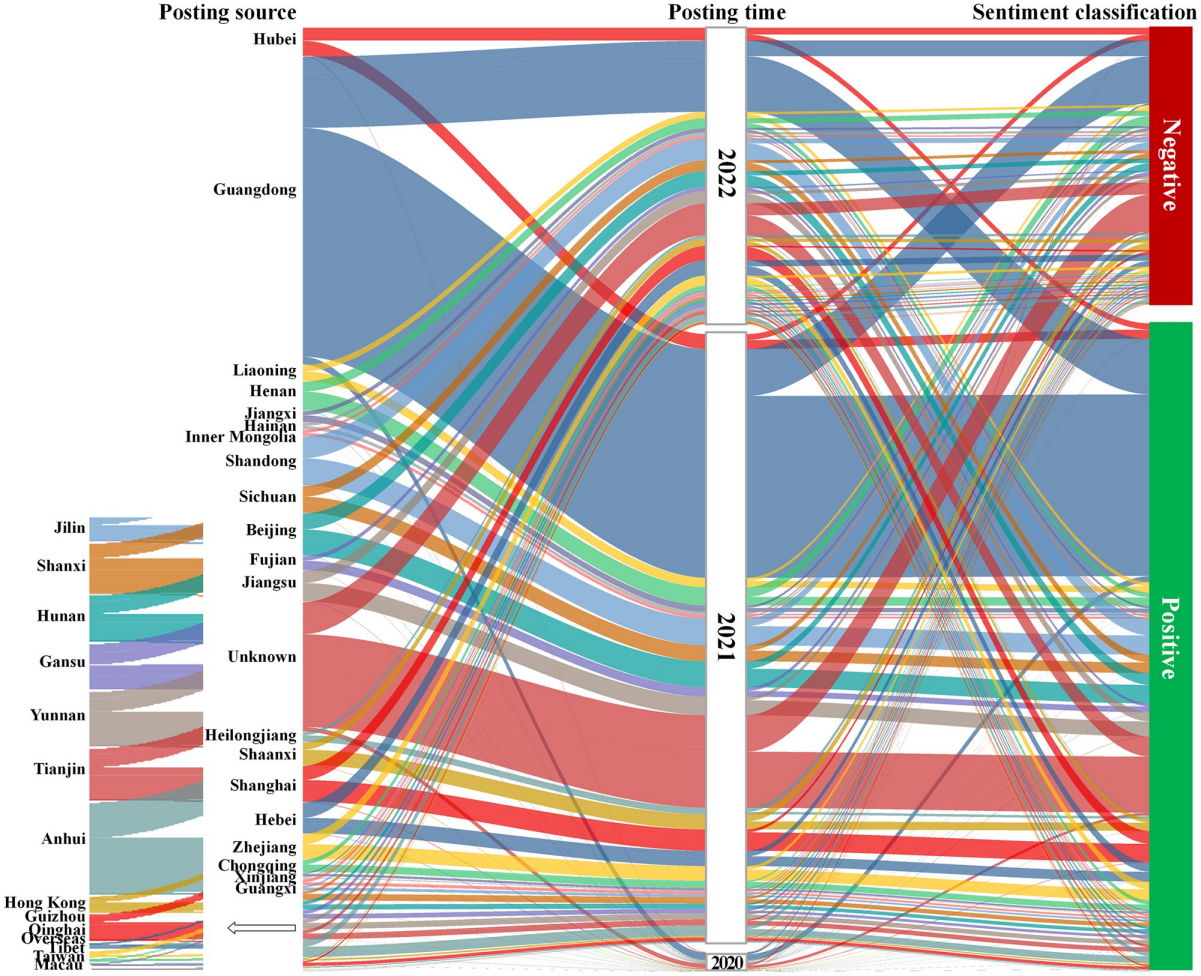
Sankey diagram of public comments characteristics on urban regeneration.

Fig 4 illustrates the results of visualizing the Sankey energy manifold graph, which presents the flow and characteristic distributions of the posting source, posting time, and sentiment classification for 29,504 public comments between December 2020 and June 2022. The width of the data stream indicates the number of comments.

First, of the 29,504 comments, 20,676 were classified as positive and 8,828 were classified as negative, resulting in a ratio of 2.34:1 of positive to negative comments and an overall average confidence level of 0.88. The results indicate that the public was supportive of urban regeneration, and overall satisfaction was high during the study period. Second, comments were posted from 37 regions, including 34 Chinese provincial administrations, Taiwan, overseas regions, and unknown regions. The number of comments and sentiment classification varied significantly across regions in different years during the study period, reflecting both spatial and temporal divergences in public commentary and sentiment toward urban regeneration.

#### Temporal distributive characteristics of public sentiment tendency

**Fig 5:**
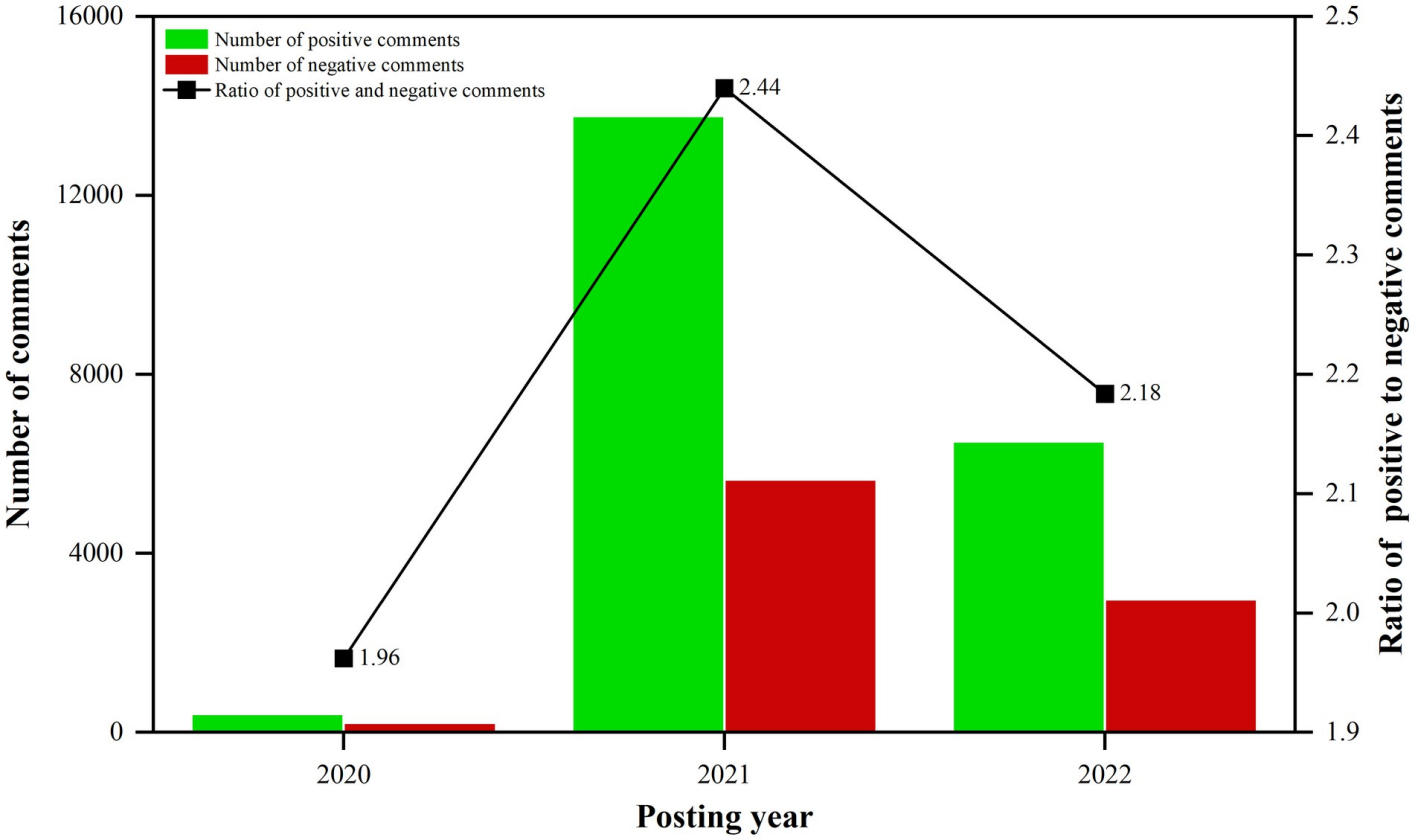
Annual variation of sentiment classification and the ratio of positive to negative comments.

First, this study evaluated general public sentiment by examining annual trends in sentiment classification results. Fig 5 illustrates the annual changes in the sentiment classification of public comments on urban regeneration and the ratio of the number of positive to negative comments during the study period. Generally, the ratio of positive to negative comments increased from 1.96:1 in 2020 to 2.44:1 in 2021, and then decreased back to 2.18:1 in 2022, suggesting that public sentiment toward urban regeneration first gradually transitioned to a higher degree of positivity, and then more negative comments were generated. Specifically, in 2020, 412 comments were classified as positive and 210 as negative, with a ratio of 1.96:1 between the number of positive and negative comments, which was lower than the overall average ratio of 2.34:1, indicating a lower degree of positive public sentiment tendency. In 2021, 13,770 comments were classified as positive and 5,644 as negative, with a ratio of 2.44:1 between the number of positive and negative comments, which was higher than the overall ratio, indicating a higher degree of positive public sentiment. In 2022, 6,494 comments were classified as positive and 2,974 as negative, with a ratio of 2.18:1 between the number of positive to negative comments, which is less than the overall ratio of 2.34:1 but greater than the ratio in 2020, indicating that public sentiment toward urban regeneration was generally more positive in 2022 than it was in 2020. This may be because before 2020, China’s urban regeneration initiatives were generally in a new situation of high-quality development [74], with fewer projects implemented and a limited renovation scale and effects, resulting in mixed public comments. In 2021, with the strong call from the central government, governments at all levels attached great importance to urban regeneration initiatives and introduced a series of implementation plans that have achieved good results and more positive public comments. Whereas, by 2022, there were more negative comments about urban regeneration due to the increase in management repair issues across numerous post-implementation urban regeneration projects, as well as the delays or even suspension of projects due to the recurrence of the SARS-CoV-2 epidemic [75].

Furthermore, to eliminate analysis bias due to differences in data volume and to quantify public sentiment tendency, this study constructed a sentiment index (SI) for each comment according to the method of Kim et al. [25]. In the case of positive content, SI = 10, whereas in the case of negative content, SI = -10. Therefore, the SI ranges from 10 to -10, with a higher SI representing a more positive sentiment, thus allowing for a comparison and assessment of sentiment tendency.

**Fig 6:**
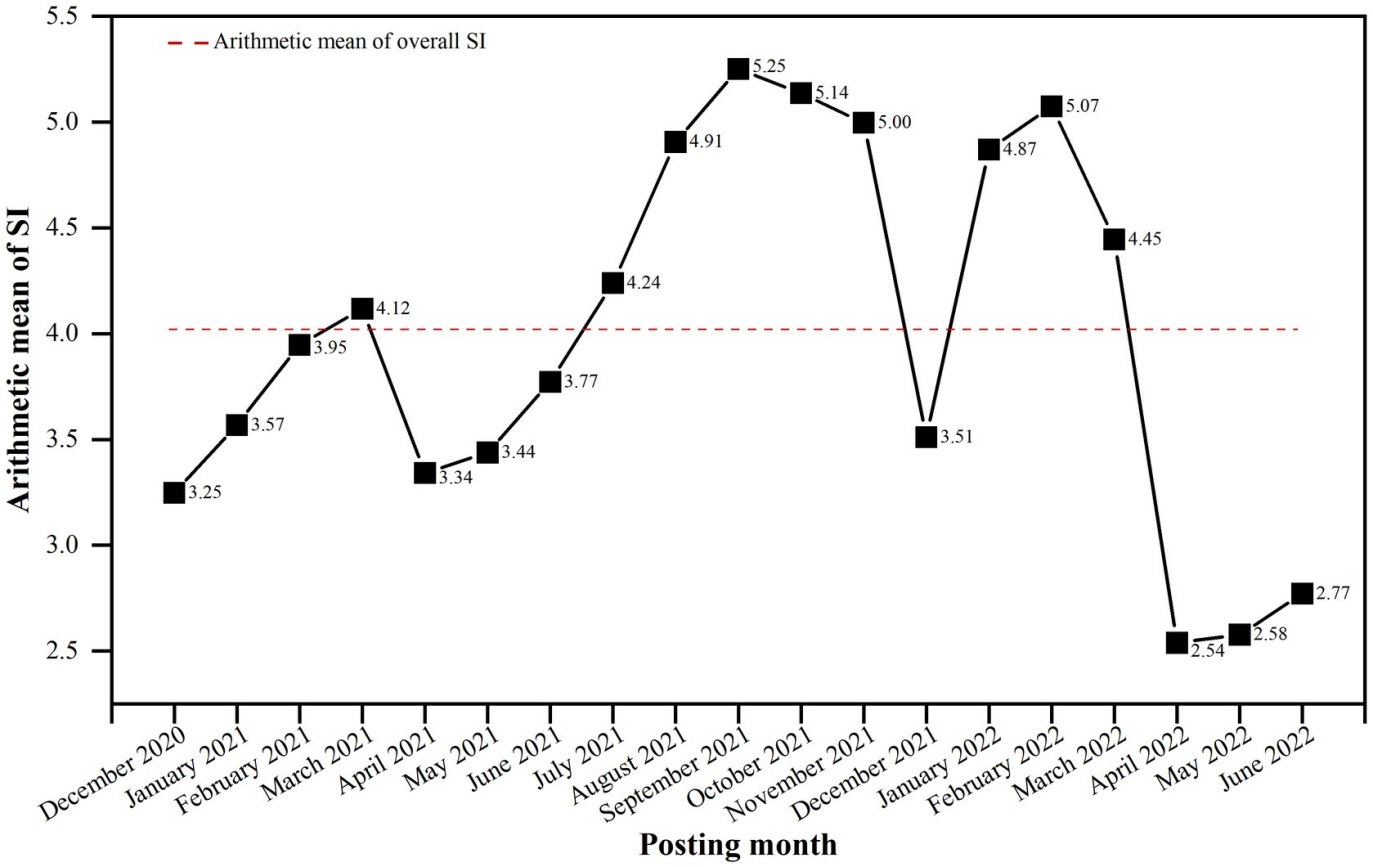
Monthly variation of arithmetic mean of SI.

To analyze the temporal characteristics of public sentiment tendency in a more segmented way, Fig 6 plots the monthly change in the arithmetic mean of the SI during the study period. The arithmetic mean of overall SI was 4.01 during the study period, indicating a general positive public sentiment tendency on urban regeneration. However, public sentiment tends to fluctuate over time. The arithmetic mean of SI continued to rise from December 2020 to March 2021 and April 2021 to September 2021, reaching a maximum of 5.25 in September 2021, indicating increasing positive public sentiment toward urban regeneration during these two periods. Despite this decrease, the arithmetic mean of SI in both periods, September to November 2021 and January to February 2022, was higher than the overall arithmetic mean of SI (4.01), indicating a more positive public sentiment of urban regeneration. After February 2022, the arithmetic mean of SI dropped to 2.54 and despite a small increase in the following three months, all remained below the arithmetic mean of the overall SI and at a low point, indicating that the public generated more negative comments regarding urban regeneration at this time. The cause of this phenomenon was the uneven results of urban regeneration projects and the recent recurrence of the SARS-CoV-2 epidemic in China [76].

#### Spatial distributive characteristics of public sentiment tendency

**Fig 7:**
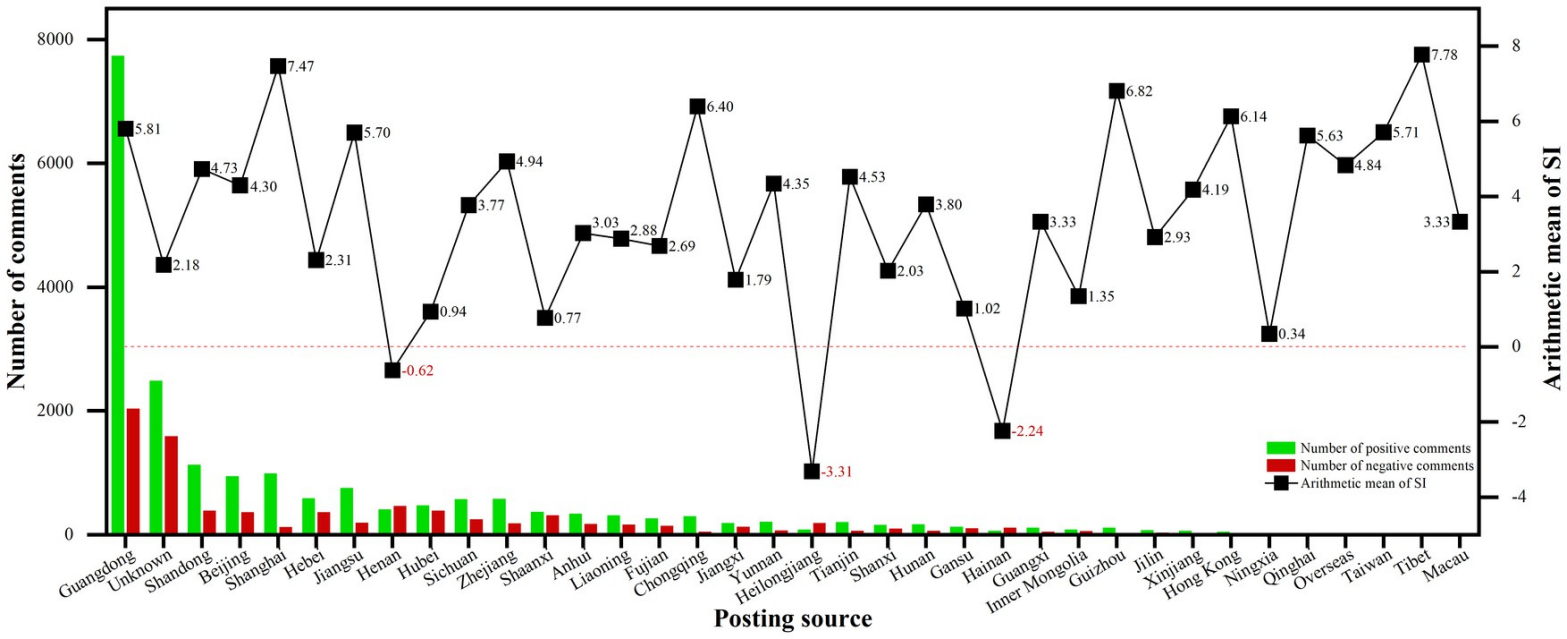
Regional distribution of sentiment classification and arithmetic mean of SI.

At the provincial level, Fig 7 displays the results of the sentiment classification and arithmetic mean SI of the 37 posting sources, which reflect differences in the number of comments and sentiment tendency on urban regeneration. In terms of the number of comments, Guangdong posted a significantly high number of comments (9,819 in total) during the study period, indicating that the public in Guangdong is more aware of urban regeneration. This may be due to the fact that the Guangdong Province, the first Chinese province to implement urban regeneration plans [77], proposed to undertake a dangerous house renovation and urban village improvement project as early as 2000. After 20 years of development, Guangdong Province introduced hundreds of urban regeneration-related policies and accumulated rich practical experience in urban regeneration, which has received wide public attention. Apart from the unknown regions, Shandong (1,561 comments), Beijing (1,351 comments), and Shanghai (1,156 comments) also have a high level of public awareness regarding urban regeneration. We observed provincial variations in sentiment tendencies. The top five provinces with high SI arithmetic means were Tibet (7.78), Shanghai (7.47), Guizhou (6.82), Chongqing (6.40), and Hong Kong (6.14), indicating that local urban regeneration initiatives have gained great social effects and public commentary. However, the arithmetic mean of SI in Heilongjiang, Hainan, and Henan was less than zero (-3.31, -2.23, and -0.62, respectively), suggesting that the public viewed local urban regeneration initiatives negatively.

**Fig 8:**
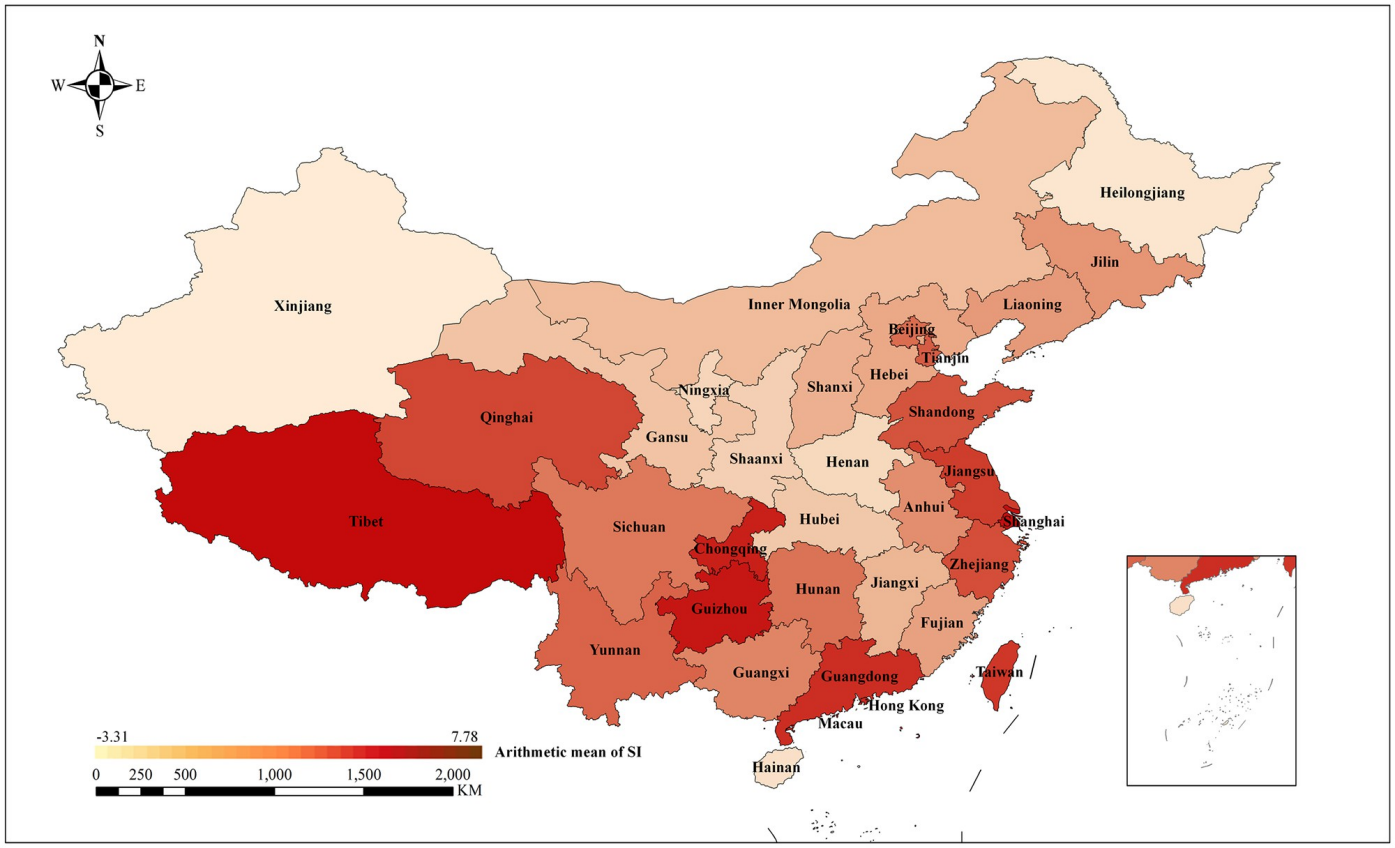
Geographical distribution of the arithmetic average of SI. Shape file source: republished from http://www.gscloud.cn under a CC BY license, with permission from Geospatial Data Cloud, original copyright [2022]; Own Map output: using ArcGIS 10.2 Software analysis.

At the national level, Fig 8 plots the geographic distribution of the arithmetic mean of SI over China (comments from unknown and overseas were excluded owing to non-locality). The darker the color, the higher the arithmetic mean of the SI in the area, showing a more positive sentiment toward local urban regeneration. Geographically, China’s eastern coast (e.g., Tianjin, Shandong, Jiangsu, Shanghai, and Zhejiang), southern coast (e.g., Guangdong and Taiwan), southwest region (e.g., Guizhou and Chongqing), and western region (e.g., Qinghai and Tibet) had high arithmetic means of SI. In contrast, the northeast (Heilongjiang, Jilin, and Liaoning), central (e.g., Inner Mongolia, Ningxia, Shaanxi, Henan, Hubei, and Jiangxi), and northwest (e.g., Xinjiang and Gansu) regions of China had low arithmetic means of SI. The spatial pattern of China’s economic development could be responsible for this phenomenon. First, the eastern and southern coastal regions of China contain three highly developed national urban agglomerations, namely the Beijing-Tianjin-Hebei urban agglomeration, the Yangtze River Delta urban agglomeration, and the Guangdong-Hong Kong-Macao Greater Bay Area [78], each of which also has favorable urban regeneration policies and open markets. There are several cities in these regions, including Shenzhen, Shanghai, and Beijing [74], which have exemplary and leading effects in the national urban regeneration field. This, along with the good results of the produced urban regeneration projects, has led to a higher level of public acceptance. Second, the southwest region of China, particularly the Chengdu-Chongqing urban agglomeration region, is the fourth pole of China’s future economic development. It is a region with great development potential that has taken practical steps to promote organic urban regeneration, which enhances the city’s sustainable growth momentum and contributes to high levels of satisfaction among its residents. Third, China’s western regions, such as Qinghai and Tibet, have witnessed a continuous net inflow of population in recent years, which has provided the foundation and development sources for urban regeneration initiatives, making them bridge cities for a new round of urban regeneration growth points. Together with the apparent positive effects of local old neighborhood renovations, public comments are generally more positive. Nevertheless, in the northeastern, central, and northwestern regions of China, which have lower levels of economic development [78], there is less support for urban regeneration policies and less effective implementation, which, in turn, results in unfavorable public appraisal.

### Public focus analysis on urban regeneration

**Fig 9:**
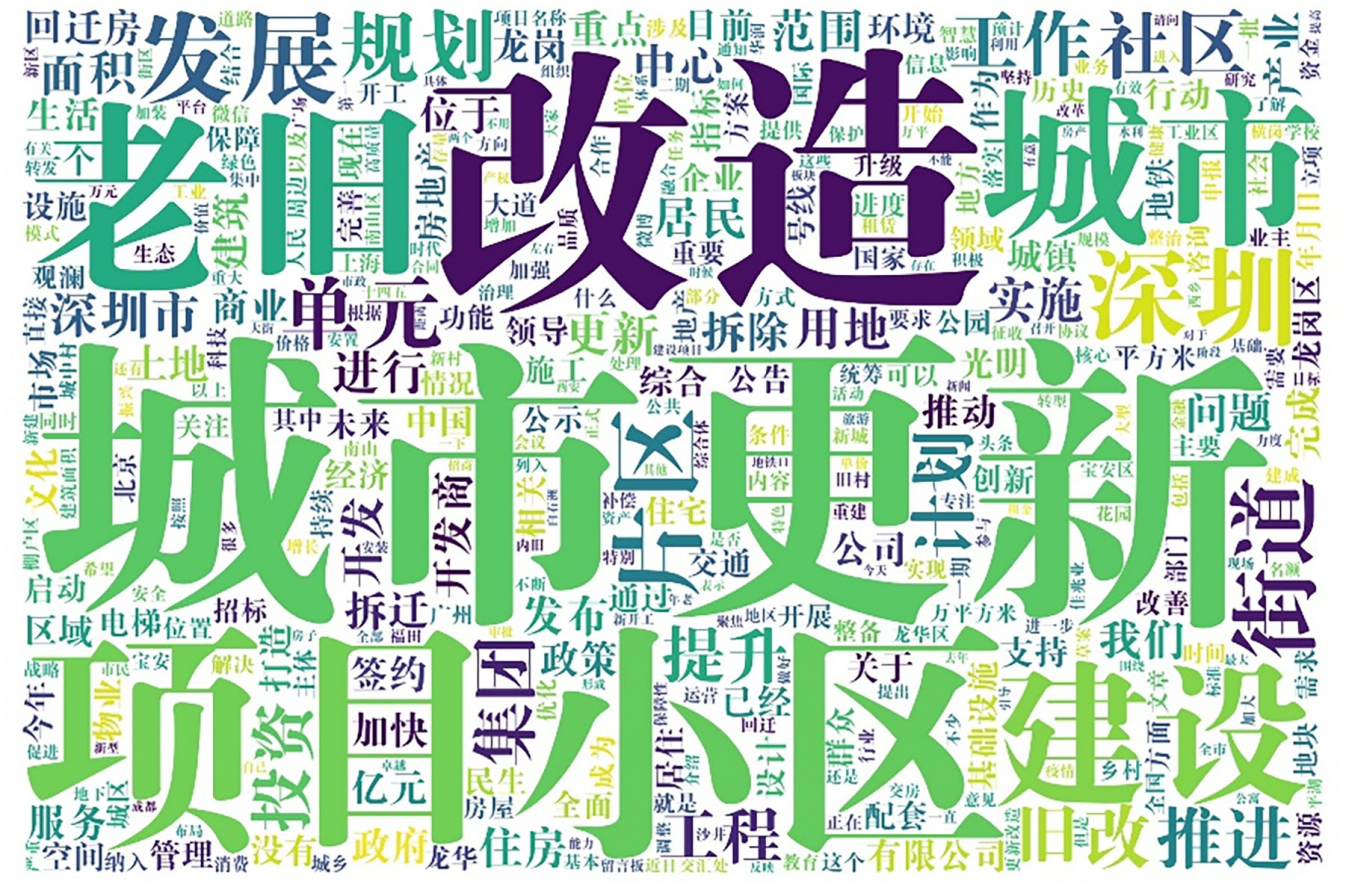
WC visualization of the top 400 most frequently posted Chinese words out of all 74,481 Chinese words.

Based on the results of the NLP-based data pre-processing and word frequency statistics, 74,481 unduplicated Chinese words were identified in all public comments. To visualize the content of the public focus on urban regeneration, Fig 9 shows the WC visualization of the top 400 most frequent Chinese words. The larger the area occupied by one Chinese word on the WC map, the more frequently it appears. The top 20 most frequent posted Chinese words, excluding the search terms, were “projects,” “urban,” “construction,” “Shenzhen,” “development,” “districts,” “streets,” “units,” “planning,” “community,” “work,” “old reform,” “advancement,” “planning,” “group,” “investment,” “upgrading,” “engineering,” “area,” and “implementation,” reflecting the public’s key focus regarding China’s urban regeneration.

### Comment topic categorization on urban regeneration

**Fig 10:**
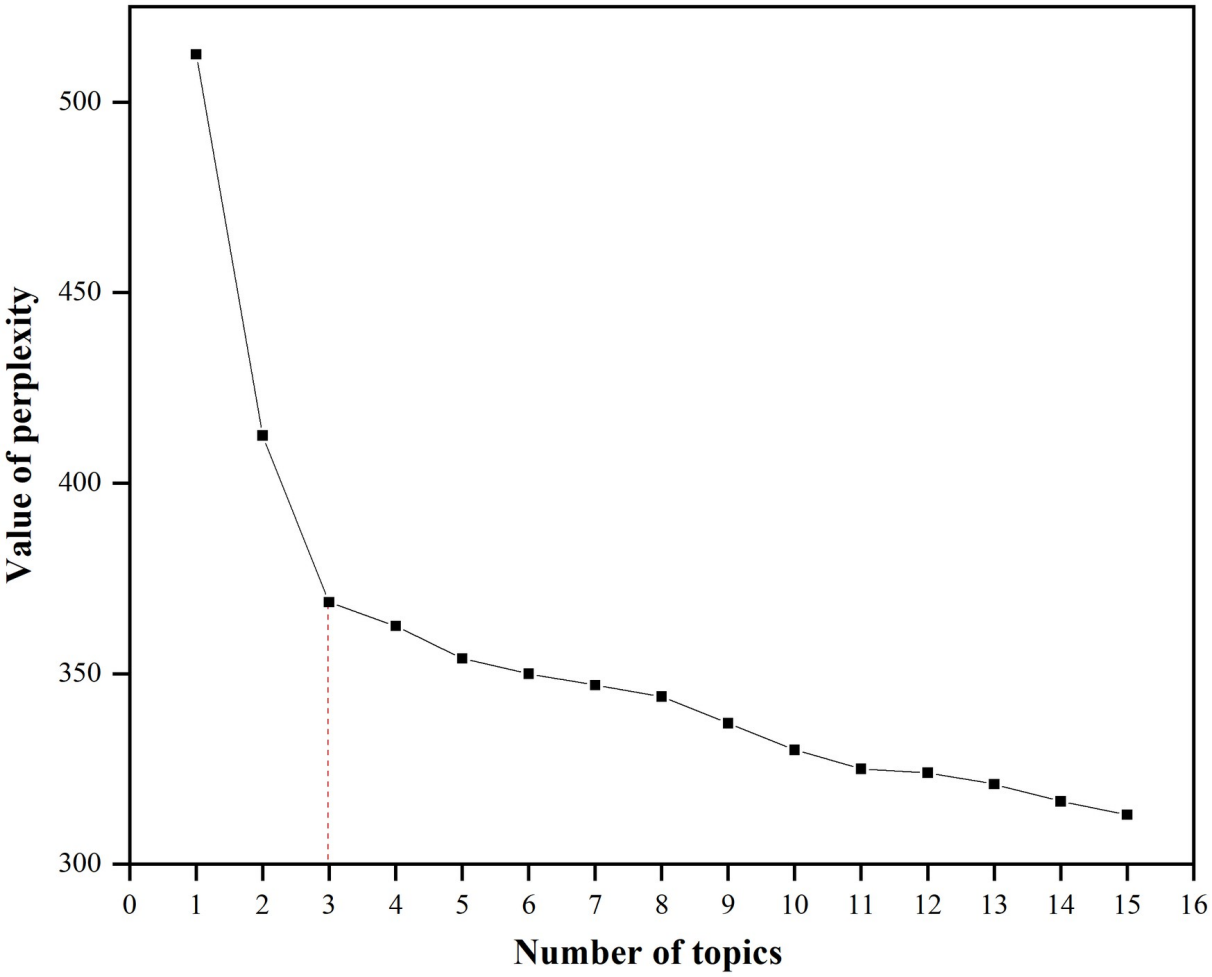
Variation in perplexity of the LDA model from 0 to 15 topics.

Fig 10 illustrates the change in perplexity of the LDA model from 0 to 15 topics. Although the greater the number of topics in an LDA model, the lower the perplexity and the greater the categorization ability of the model, an excessive number of topics may lead to overfitting. Therefore, in conjunction with the elbow method, which determines the optimal number of topics based on the inflection point during the change in perplexity, this study regarded three as an optimal number of topics.

**Fig 11:**
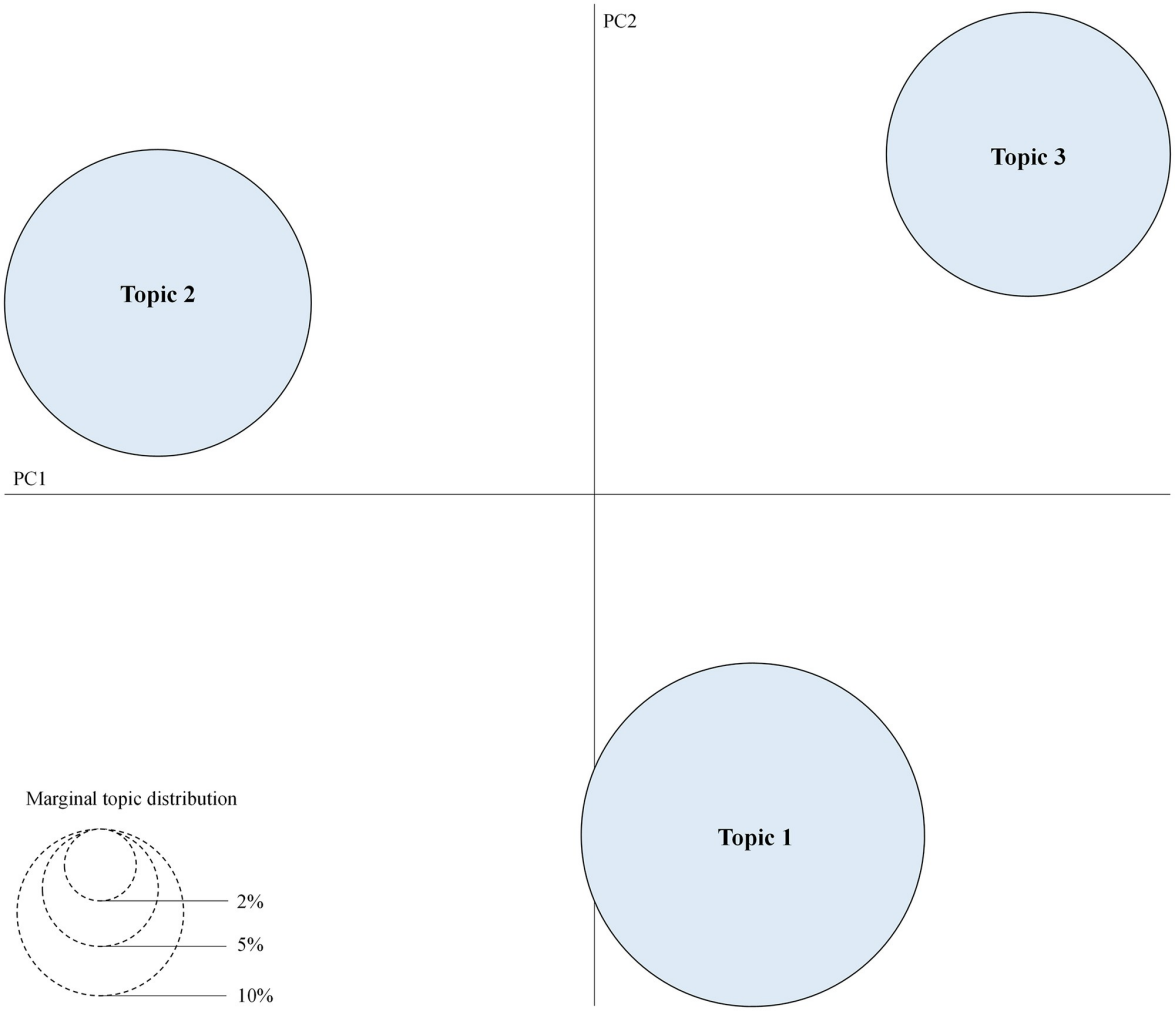
IDM of the three clusters categorized using the LDA model.

Having categorized 29,504 comments into three topics using the LDA model, the IDM was required to verify the validity of the clustering results. As shown in Fig 11, the three clusters are graphically uncrossed and distributed far apart, thereby demonstrating the high validity of the LDA model.

Consequently, 29, 504 comments were categorized into three topics based on the LDA model. Table 2 presents the results of the LDA model for the topic categorization and the words included in each topic (words that occur more predominantly contribute more to the categorization of its topic). The first topic contained terms such as "Shenzhen," "Group," "Area," "Plan," and "Developer," which pertain to the urban regeneration and old renovation of Shenzhen. It is most probably due to the fact that, as a sub-provincial city of Guangdong Province, Shenzhen is a pioneer in the construction of China’s urban regeneration system [79] and therefore received high public concern. This is consistent with the findings on the spatial distribution characteristics of sentiment tendency. The second topic comprised words such as "Development," "Investment," "Industry," "Promotion," and "Billion," which are related to the development and strategic planning of urban regeneration at a general level, reflecting public concerns about urban regeneration at a macro level. The third topic addressed a range of issues and problems that urban regeneration poses to its residents on a micro level and focused on "Neighborhoods," "Residents," "Problems," "Community," "Leadership," "Lifts," and "Town." A majority of these negative words were posted on message boards or city forums, reflecting the public’s complaints regarding urban micro-regeneration. Notably, the word "Lift" reflects the widespread attention given to the retrofitting of lifts for old neighborhoods.

**Table 2:** Topic categorization of all comments based on the LDA model.

Topic	Words Contained (top 15)
Topic 1	"Urban regeneration," "Project," "Shenzhen," "District," "Unit," "Street," "Group," "Engineering," "Area," "Planning," "Land use," "Scope," "Plan," "Located," "Developer"
Topic 2	"Urban regeneration," "Urban," "Construction," "Development," "Projects," "Promote," "Investment," "Upgrade," "Industry," "Implementation," "Work," "Housing," "Accelerate," "Promotion," "Billion"
Topic 3	"Improvement," "Neighborhoods," "Residents," "Problems," "Works," "We," "Carry out," "Projects," "Community," "Work," "No," "Leadership," "Plans," "Lifts," "Town"

## Discussion and conclusion

This study performs a sentiment analysis of 29,504 public comments on China’s urban regeneration from December 2020 to June 2022 by using a combination of NLP, an advanced DL architecture-SKEP, WC and an advanced traditional ML model-LDA. In comparison to previous studies on public sentiment analysis pertaining to urban regeneration [31–33] and old industrial renovation [16–19], this study is the first to use massive data collected from social media, online forums, and government affairs platforms to analyze public sentiments. The aim of this study is to objectively and comprehensively investigate people’s true will and sentiments regarding China’s urban regeneration. The results demonstrate the social benefits of urban regenerations and provide a realistic basis and macro reference for the development of China’s urban regeneration based on its ultimate goal of satisfying people’s long-cherished wish for a better life. We suggest that governments and practitioners should address spatiotemporal disparities and concerns of local residents in public support for future development of China’s urban regeneration initiatives. The main findings of this study are as follows:

1. SKEP model has demonstrated the highest accuracy, precision, recall, and F1-score in sentiment classification tasks compared to BERT model and the current state-of-the-art RoBERTa model.
2. A general positive public sentiment toward China’s urban regeneration has been confirmed, with an overall ratio of positive to negative comments of 2.3:1 and an arithmetic mean of SI of 4.01 (under an overall confidence level of 0.88). In comparison with previous studies that conducted local and web questionnaire surveys on specific renovation projects [16, 17, 32], our results appear to be more pessimistic. This may be due to the fact that we used a more comprehensive and extensive dataset, and more complaints and questioning were revealed. Moreover, spatiotemporal divergences in public sentiment have been observed.
  - Temporally, public sentiment tends to be most negative in 2020, most positive in 2021, and declining in 2022, especially after February 2022. This phenomenon can be attributed to the gradual improvement in the effectiveness of urban regeneration projects in China from an initial launch in 2020 to a full-scale promotion in 2021, wherein more positive comments were generated. However, in 2022, as a result of the SARS-CoV-2 lockdown in China, which results in a conflict between the increasing number of management repair issues and the suspension of renovation projects, more negative public commentary was generated.
  - Spatially, at the provincial level, Guangdong, the first region in China to implement urban regeneration plans [77], posted the most comments, showing considerable interest in urban regeneration. Further, Tibet, Shanghai, Guizhou, Chongqing, and Hong Kong are the top five provinces with highly positive public sentiment toward local urban regeneration. At the national level, China’s east and south coastal regions, southwest region, and western region tend to be more positive toward urban regeneration, while those in the northeast, central, and northwest regions tend to be more negative. Several factors can explain this phenomenon, including divergent regional economic development and different policy supports for local urban regeneration.
3. The key focus of the public on China’s urban regeneration includes "projects," "urban," "construction," "Shenzhen," "development," "districts," "streets," "units," "planning," "community," "work," "old reform," "advancement," "planning," "group," "investment," "upgrading," "engineering," "area," and "implementation."
4. Public comments are categorized into three topics. The first topic relates to urban regeneration and old renovation in Shenzhen, which coordinates the key focus of the public. This be explained by its leading position in China’s urban regeneration [79]. The second topic concerns the development of the urban regeneration sector and the strategic planning of urban regeneration, evidencing the public’s interest at a macro level. "Investment," "Upgrade," "Industry," "Implementation," and "Work," in this topic have also been the public’s key focus. The third topic deals with the problems and issues experienced by residents due to the regeneration initiatives, especially the issue of lift retrofitting for old neighborhoods, which represents the current deficiencies and shortcomings experienced by China’s urban regeneration schemes at the micro level. In this topic, "Projects," "Community," "Work," have also been confirmed to be the public focus.

## Limitation

This study has some limitations. First, this study attempted to capture as many public comments as possible from Weibo, 466 online forums, and 42 government affairs platforms from December 2020 to June 2022. However, due to anti-crawler settings and manual verification of various websites, the number of comments collected by the study is restricted and time-consuming. Furthermore, since the study used online data, the comments may not be representative of the entire Chinese public, particularly those who are not active online. Moreover, residents generally complain when responding to online questionnaires [80], which increases negative comments. Therefore, the arithmetic mean of the SI may be underestimated. As part of future research, comments could be obtained from a variety of online and offline sources over a longer period to examine diverse public sentiments regarding urban regeneration.

Second, despite using the current state-of-the-art sentiment classification model SKEP for the binary classification of public comments (positive or negative), this study does not classify comments that are neutral, which may result in an increased variance and reduced stability of the sentiment classification results. Further research could focus on classifying sentiments in more detail, including ternary sentiment classification (negative, neutral, and positive) as well as multiple sentiment classification (varying degrees of negativity and positivity).

Third, although this study offers a qualitative explanation for the different spatial distributions of sentiment tendencies based on divergent regional economic development and policies, it lacks a quantitative explanation. Future research could explore the relationship between regional economic and public sentiment tendencies on urban regeneration using quantitative spatial correlation indicators such as the Moran Index [81].

Fourth, in terms of topic categorization, this study clusters and extracts features from three topics of comment content using the currently advanced LDA model, but the resulting topics still require human identification and adoption, increasing the degree of bias and ambiguity by human intervention.

## Supporting information

S1 Table 466 Chinese forum websites and 42 Chinese government affairs platforms. (DOCX)

S2 Table 41,248 collected comments. (XLSX)

S1 File Analysis code. (ZIP)

## Abbreviations

BERT: Bidirectional Encoder Representations from Transformers
DL: Deep Learning
IDM: Inter-Topic Distance Map
LDA: Latent Dirichlet Allocation
ML: Machine Learning
NLP: Natural Language Processing
RoBERTa: Robustly optimized Bidirectional Encoder Representations from Transformers
SI: Sentiment Index
SKEP: Sentiment Knowledge Enhanced Pre-training
WC: Word Cloud
