# Supplementary material for: Public sentiment analysis on urban regeneration: A massive data study based on sentiment knowledge enhanced pre-training and latent Dirichlet allocation
Source: PLoS One. 2023 Apr 27;18(4):e0285175. doi: 10.1371/journal.pone.0285175 (PMC10138235; doi:10.1371/journal.pone.0285175)
Supplement: S1 Table — (DOCX) [file pone.0285175.s001.docx]

**Supporting information**

**S1 Table. 466 Chinese forum websites and 42 Chinese government affairs platforms.**

| **Data sources (original in Chinese)** | **Data sources (translated in English)** | **Attribute** |
| --- | --- | --- |
| 12345上海市民服务热线 | 12345-Shanghai Citizen Service Hotline | Government affairs platform |
| 问政四川网 | Ask Sichuan Website | Government affairs platform |
| 成都市人民政府网络理政平台 | Chengdu People's Government Web-based Government Platform | Government affairs platform |
| 中国·平凉门户网站留言 | China-Pingliang Portal Message | Government affairs platform |
| 重庆网络问政平台 | Chongqing Network Q&A Platform | Government affairs platform |
| 大同市12345政府服务热线 | Datong 12345 Government Service Hotline | Government affairs platform |
| 丰城网络问政 | Fengcheng Web Government Platform | Government affairs platform |
| 福州市12345便民服务平台 | Fuzhou 12345 Convenient People Service Platform | Government affairs platform |
| 福州市12345政府公共服务系统 | Fuzhou 12345 Government Public Service System | Government affairs platform |
| 海口市政府12345热线 | Haikou 12345 Government Hotline | Government affairs platform |
| 海口网问政海口 | Haikou Website Ask Politics Haikou | Government affairs platform |
| 海南省政府12345热线 | Hainan Provincial Government 12345 Hotline | Government affairs platform |
| 哈尔滨市人民政府 | Harbin People's Government | Government affairs platform |
| 红网问政湖南 | Hong Website: Ask the Hunan Government | Government affairs platform |
| 惠州市人民政府 | Huizhou People's Government | Government affairs platform |
| 葫芦岛市12345政府服务热线 | Huludao 12345 Government Service Hotline | Government affairs platform |
| 百湖民声大庆市网络问政平台 | Hundred Lakes Min Sheng Daqing City Online Government Platform | Government affairs platform |
| 荆门市委书记专属版 | Jingmen Municipal Party Secretary Exclusive Edition Website | Government affairs platform |
| 六安市金安区人民政府 | Liuan Jinan District People's Government | Government affairs platform |
| 泸州网络问政平台 | Luzhou network questioning platform | Government affairs platform |
| 南阳市长网上留言板 | Nanyang Mayor Online Message Board | Government affairs platform |
| 南阳书记网上留言板 | Nanyang Secretary Online Message Board | Government affairs platform |
| 人民网地方领导留言板 | People Website: Local Leaders Message Board | Government affairs platform |
| 平潭综合实验区12345政务服务平台 | Pingtan Comprehensive Experimental Zone 12345 Government Service Platform | Government affairs platform |
| 青岛政务网 | Qingdao Government Website | Government affairs platform |
| 三明市12345便民服务平台 | Sanming 12345 Convenient Service Platform | Government affairs platform |
| 三亚12345政府服务热线 | Sanya 12345 Government Service Hotline | Government affairs platform |
| 汕头市12345政府服务热线 | Shantou 12345 Government Service Hotline | Government affairs platform |
| 十堰市人民政府 | Shiyan People's Government | Government affairs platform |
| 阳光热线问政平台 | Sunshine Hotline | Government affairs platform |
| 泗阳12345政务服务热线 | Suzhou 12345 Government Service Hotline | Government affairs platform |
| 天水市委书记、市长留言板 | Tianshui Municipal Party Secretary and Mayor Message Board | Government affairs platform |
| 通衢网络问政平台 | Tongqu Network Q&A Platform | Government affairs platform |
| 万州问政网络平台 | Wanzhou Q&A Network Platform | Government affairs platform |
| 温州市网络问政平台 | Wenzhou Online Government Platform | Government affairs platform |
| 武汉城市留言板 | Wuhan City Message Board | Government affairs platform |
| 邢台市综合网络问政平台 | Xingtai Comprehensive Network Q&A Government Platform | Government affairs platform |
| 零距离问政 | Zero Distance Ask Politics | Government affairs platform |
| 漳州市12345便民服务平台 | Zhangzhou 12345 Convenient Service Platform | Government affairs platform |
| 湛江市12345市民服务热线 | Zhanjiang 12345 Citizen Service Hotline | Government affairs platform |
| 政民零距离 | Zhengminglingjuli Website | Government affairs platform |
| 郑州心通桥 | Zhengzhou Xin Tong Qiao Website | Government affairs platform |
| 108社区 | 108-Community | Forum website |
| 315质量曝光 | 315-Quality Exposure Website | Forum website |
| 52庐江论坛 | 52-Lujiang Forum | Forum website |
| 5熊猫 | 5-Panda Website | Forum website |
| 爱卡汽车论坛 | Aika Auto Forum | Forum website |
| 爱卡汽车 | Aika Auto Website | Forum website |
| 阿拉宁波网 | AlaNingbo Website | Forum website |
| 安阳论坛 | An Yang Forum | Forum website |
| 安庆e网 | Anqing eNet Website | Forum website |
| 安庆论坛 | Anqing Forum | Forum website |
| 安乡论坛 | Anxiang Forum | Forum website |
| 奥一报料 | Aoyi News | Forum website |
| 奥一网 | Aoyi Website | Forum website |
| 问政抚州 | Ask Fuzhou | Forum website |
| 问政赣州 | Ask Ganzhou | Forum website |
| 问政江西 | Ask Jiangxi | Forum website |
| 问政深圳 | Ask Shenzhen | Forum website |
| 问政四川 | Ask Sichuan | Forum website |
| 问政唐山 | Ask Tangshan | Forum website |
| 问政鹰潭 | Ask Yingtan | Forum website |
| 问政永州 | Ask Yongzhou | Forum website |
| 巴蜀论坛 | Ba Shu Forum | Forum website |
| 百度知道 | Baidu Know Website | Forum website |
| 百度贴吧 | Baidu Posting Website | Forum website |
| 芭乐网 | Bale Website | Forum website |
| 宝宝树孕育 | BaoBao Tree Website | Forum website |
| 包头市公安局 | Baotou City Public Security Bureau | Forum website |
| 蜂巢物业论坛 | Beehive Property Forum | Forum website |
| 北方网 | BeiFang Website | Forum website |
| 北海365 | Beihai365 Forum | Forum website |
| 北海365网 | Beihai365 Website | Forum website |
| 蚌埠论坛 | Bengbu Forum | Forum website |
| 冰城网论坛 | Bingcheng Website Forum | Forum website |
| 滨海论坛 | Binhai Forum | Forum website |
| 黑猫投诉 | Black Cat Complaints | Forum website |
| 博客中国 | Blog China | Forum website |
| 亳州生活网 | Bozhou Life Website | Forum website |
| 釜溪论坛 | Buxi Forum | Forum website |
| 蔡甸在线 | Caidian Online | Forum website |
| 彩龙社区 | Cailong Community | Forum website |
| 财新网 | Caixin Website | Forum website |
| 查查网 | Chacha Website | Forum website |
| 长城网 | Changcheng Website | Forum website |
| 常德论坛 | Changde Forum | Forum website |
| 长葛网 | Changge Website | Forum website |
| 长乐论坛 | Changle Forum | Forum website |
| 长沙社区通 | Changsha Community | Forum website |
| 长兴岛论坛 | Changxing Island Forum | Forum website |
| 长兴在线 | Changxing Online | Forum website |
| 巢湖社区论坛 | Chaohu Community Forum | Forum website |
| 茶竹永川论坛 | Chazhu Yongchuan Forum | Forum website |
| 成都论坛 | Chengdu Forum | Forum website |
| 成都向上 | Chengdu Up Website | Forum website |
| 赤壁论坛 | Chibi Forum | Forum website |
| 赤脚论坛 | Chijiao Forum | Forum website |
| 中国会计视野论坛 | China Accounting Horizons Forum | Forum website |
| 中国企业员工互动论坛 | China Enterprise Staff Interactive forum | Forum website |
| 中国洛阳 | China Luoyang Website | Forum website |
| 中国推广网 | China Promotion Website | Forum website |
| 中国证券网博客 | China Securities Blog | Forum website |
| 池州人论坛 | Chizhou People Forum | Forum website |
| 池州查查网 | Chizhouchacha Website | Forum website |
| 池州人 | Chizhouren Website | Forum website |
| 重庆论坛 | Chongqing Forum | Forum website |
| 重庆购物狂 | Chongqing Shopaholic Website | Forum website |
| 稠州论坛 | Chouzhou Forum | Forum website |
| 创幻财经 | Chuang Phantom Finance | Forum website |
| 滁州论坛 | Chuzhou Forum | Forum website |
| 城市联盟 | CityUnion Website | Forum website |
| 慈溪论坛 | Cixi Forum | Forum website |
| 慈溪网 | Cixi Website | Forum website |
| CSDN技术社区 | CSDN Technical Community | Forum website |
| 大邯郸 | DaHandan Website | Forum website |
| 大河论坛 | Dahe Forum | Forum website |
| 宽带山社区 | Daikuan Shan Community | Forum website |
| 宽带山 | Daikuan Shan Website | Forum website |
| 大头菜论坛 | Daitoucai Forum | Forum website |
| 大江论坛 | Dajiang Forum | Forum website |
| 大阆网 | Dalang Website | Forum website |
| 当涂ok论坛 | Dangtuok Forum | Forum website |
| 当阳热线 | Danyang Hotline | Forum website |
| 丹阳翼网 | Danyang Wing Network | Forum website |
| 大濮网 | Dapu Website | Forum website |
| 大千社区 | Daqian Community | Forum website |
| 大亚湾在线 | Dayawan Online | Forum website |
| 大宜宾 | Dayibin Website | Forum website |
| 大舟山论坛 | Dazhoushan Forum | Forum website |
| 德清108社区 | Deqing 108 Community | Forum website |
| 得意生活 | Deyishenghuo Website | Forum website |
| 发现荆门 | Discover Jingmen | Forum website |
| 东方论坛 | Dongfang Forum | Forum website |
| 东方热线 | Dongfang Hotline | Forum website |
| 东风论坛 | Dongfeng Forum | Forum website |
| 东莞阳光网 | Dongguan Sunshine Network | Forum website |
| 东林书院 | Donglinshuyuan Website | Forum website |
| 豆瓣 | Douban Website | Forum website |
| 都昌在线 | Duchang online | Forum website |
| 渠县网 | Duxian Website | Forum website |
| 东湖社区 | East Lake Community | Forum website |
| 东太湖论坛 | East Taihu Forum | Forum website |
| e滁州社区 | E-Chuzhou Community | Forum website |
| e滁州网 | E-Chuzhou Website | Forum website |
| 生态梦 | Ecological Dream Website | Forum website |
| 二千沙龙社区 | Erqian salon community | Forum website |
| 二泉网-无锡论坛 | Erquan Website - Wuxi Forum | Forum website |
| 鄂州一网 | Ezhou net | Forum website |
| 法律问答 | Falv Q&A | Forum website |
| 法律快车 | Falvkuaiche Website | Forum website |
| 房天下 | Fangtianxia Website | Forum website |
| 方竹论坛 | Fangzhu Forum | Forum website |
| 法妞问答 | Faniu Q&A | Forum website |
| 奉化论坛 | Feng Hua Forum | Forum website |
| 凤凰山下 | Fenghuangshanxia Website | Forum website |
| 凤台小鱼网 | Fengtaixiaoyu Network | Forum website |
| 风闻社区 | Fengwen Community | Forum website |
| 丰县论坛 | Fengxian Forum | Forum website |
| 金融界 | Financial Community | Forum website |
| 菲龙网 | Firong Website | Forum website |
| 福步外贸论坛 | Fubu Foreign Trade Forum | Forum website |
| 涪陵在线 | Fuling Online | Forum website |
| 涪风论坛 | Fulwind Forum | Forum website |
| 阜宁网 | Funing Website | Forum website |
| 富顺在线 | Fushun Online | Forum website |
| 富阳19楼 | Fuyang19F Website | Forum website |
| 富裕信息网 | Fuyu Information Network | Forum website |
| 赣问 | Ganwen Website | Forum website |
| 高古楼 | Gaogulou Website | Forum website |
| 高楼迷 | Gaokloumi Website | Forum website |
| 高密信息网 | Gaomi Information Network | Forum website |
| 高明论坛 | Gaoming Forum | Forum website |
| 全球征集网 | Global Collection Network | Forum website |
| 灌南论坛 | Gounan Forum | Forum website |
| 广德论坛 | Guangde Forum | Forum website |
| 广饶信息网 | Guangrao Information Network | Forum website |
| 广州妈妈网 | Guangzhoumama Website | Forum website |
| 固安圈 | Gu'anquan Website | Forum website |
| 股吧 | Guba Website | Forum website |
| 贵港新闻网 | Guigang News | Forum website |
| 桂林人论坛 | Guilin People's Forum | Forum website |
| 股票之声 | Gupiaozhisheng Website | Forum website |
| 海安零距离 | Hai'an Zero Distance | Forum website |
| 化海川流海川化工论坛 | Haichuan Chemical Forum | Forum website |
| 海安网 | Haikou Website | Forum website |
| 海南大学论坛 | Hainan University Forum | Forum website |
| 海棠社区 | Haitang Community | Forum website |
| 杭州19楼论坛 | Hangzhou 19 Floor Forum | Forum website |
| 杭州 | Hangzhou Website | Forum website |
| 汉中在线 | Hanzhong Online | Forum website |
| 濠滨论坛 | Haobin Forum | Forum website |
| 好向圈 | Haoxiangquan Website | Forum website |
| 濠友 | Haoyou Website | Forum website |
| 河北新闻网-阳光理政 | Hebei News - Sunshine Politics | Forum website |
| 合浦论坛 | Hepu Forum | Forum website |
| 合浦123论坛 | Hepu123 Forum | Forum website |
| 合优网 | Heyou Website | Forum website |
| 家在深圳 | Home in Shenzhen Website | Forum website |
| 红网消费维权 | Hong Website: Consumer Rights | Forum website |
| 红豆社区 | Hongdou Community | Forum website |
| 红网论坛 | Hongwang Forum | Forum website |
| 红网百姓呼声 | Hongwang People's Voice | Forum website |
| 洪雅论坛 | Hongya Forum | Forum website |
| 洪泽论坛 | Hongze Forum | Forum website |
| 淮北人网 | Huabeiren Website | Forum website |
| 淮北人 | Huaibeiren Website | Forum website |
| 淮水安澜 | Huaishuianlan Website | Forum website |
| 怀远论坛 | Huaiyuan Forum | Forum website |
| 华律网 | Hualu Website | Forum website |
| 黄冈信息网 | Huangganginfo Website | Forum website |
| 黄岩论坛 | Huangyan Forum | Forum website |
| 华商论坛 | Huashang forum | Forum website |
| 华声在线湘问投诉直通车 | Huasheng Online Xiangqian Complaints Direct | Forum website |
| 湖北新视点 | Hubei New Viewpoint | Forum website |
| 化龙巷 | Huilongxiang Website | Forum website |
| 胡集论坛 | Huji Forum | Forum website |
| 百姓书记交流平台 | Hundreds of Secretaries Exchange Platform | Forum website |
| 霍邱网 | Huoqiu Website | Forum website |
| 虎扑社区 | Huwu Community | Forum website |
| 虎嗅网 | Huxiu Website | Forum website |
| 江山多娇户外旅游网 | Jiangshanduojiaooutdoortravel Website | Forum website |
| 江夏tv | Jiangxiatv Website | Forum website |
| 建阳论坛 | Jiangyang Forum | Forum website |
| 胶东在线 | Jiaodong Online | Forum website |
| 嘉鱼热线 | Jiaoyu Hotline | Forum website |
| 嘉兴人论坛 | Jiaxingman Forum | Forum website |
| 揭阳星空网 | Jieyangxingkong Website | Forum website |
| 京华社区 | Jinghua Community | Forum website |
| 金犍为 | Jingjianwei Website | Forum website |
| 荆门社区 | Jingmen community | Forum website |
| 荆州新闻网 | Jingzhounews Website | Forum website |
| 金湖论坛 | Jinhu Forum | Forum website |
| 金华论坛 | Jinhua Forum | Forum website |
| 锦州新闻网 | Jinzhou News | Forum website |
| 九江论坛 | Jiujiang Forum | Forum website |
| 九曲河门户网 | Jiuquhe Website | Forum website |
| 君安乐园网 | JunanLand Website | Forum website |
| 军转网 | Junzhuan Website | Forum website |
| 看福清 | Kanfuqing Website | Forum website |
| 看靖江 | Kanjingjiang Website | Forum website |
| 客家新闻网 | Kejia News | Forum website |
| 克米设计 | Kemi Design Website | Forum website |
| 凯里人论坛 | Kerryman Forum | Forum website |
| 科学网 | Kexue Website | Forum website |
| 口水杭州 | Koushui Hangzhou Website | Forum website |
| 昆山论坛 | Kunshan Forum | Forum website |
| 瓢城网 | Ladybird Website | Forum website |
| 莱芜论坛 | Laiwu Forum | Forum website |
| 莱芜在线 | Laiwu Online | Forum website |
| 老虎社区 | Laohu Community | Forum website |
| 遗爱网 | Legacy Website | Forum website |
| 临汾365网 | Lianfen365 Website | Forum website |
| 梁溪网 | Liangxi Website | Forum website |
| 辽宁社区 | Liaoning Community | Forum website |
| 篱笆网 | Liba Website | Forum website |
| 生活圈 | Life Circle Website | Forum website |
| 临安19楼 | Linan19F Website | Forum website |
| 灵璧论坛 | Lingbi Forum | Forum website |
| 响水网 | Lingshui Website | Forum website |
| 临清在线 | Linqing Online | Forum website |
| 溧水114网 | Lishui114 Website | Forum website |
| 六安城市网 | Liuan City Network | Forum website |
| 六安论坛 | Liuan Forum | Forum website |
| 六安政府网百姓畅言 | Liuan Government: People Speak Freely | Forum website |
| 六安人客户端 | Liuanren Client | Forum website |
| 六安人论坛 | Liuanren Forum | Forum website |
| 理想论坛 | Lixiang Forum | Forum website |
| 溧阳论坛 | Liyang Forum | Forum website |
| 龙城博客 | Longcheng blog | Forum website |
| 龙岩kk网 | Longyankk Website | Forum website |
| 爱我环保学社 | Love Me Environmental Learning Society | Forum website |
| 爱威海社区 | Love Weihai Community | Forum website |
| 爱上城口 | Lovechengkou Website | Forum website |
| 爱洛阳网 | LoveLuoyang Website | Forum website |
| 罗湖社区家园网论坛 | Luohu Community Home Network Forum | Forum website |
| 罗湖社区家园网 | Luohucommunity Website | Forum website |
| 罗塘人家 | Luotangjia | Forum website |
| 罗田论坛 | Luotian Forum | Forum website |
| 洛阳信息港 | Luoyang Information Port | Forum website |
| 洛阳网:百姓呼声 | Luoyang People's Voice | Forum website |
| 洛阳网 | Luoyang Website | Forum website |
| 泸州新闻网-报料 | Luzhou News Network-Reporting | Forum website |
| 泸州在线 | Luzhou online | Forum website |
| 泸州小蚂蚁 | Luzhouxiaomayi | Forum website |
| 马鞍山市民心声论坛 | Ma On Shan Citizen's Voice Forum | Forum website |
| 马鞍山OK论坛 | Maanshan OK Forum | Forum website |
| 马蜂窝 | Mafengwo Website | Forum website |
| 麦地网 | Maidi Website | Forum website |
| 麻辣社区 | Mala Community | Forum website |
| 妈妈网 | Mama Website | Forum website |
| 茂名论坛 | Maoming Forum | Forum website |
| 茂名传媒网 | Maoming Media Network | Forum website |
| 五月天论坛 | Mayday Forum | Forum website |
| 眉山人论坛 | Meishanren Forum | Forum website |
| 眉山人网 | Meishanren Website | Forum website |
| 梅州时空论坛 | Meizhoushikong forum | Forum website |
| 梦溪论坛 | Mengxi Forum | Forum website |
| 民生e点通 | Minsheng eDotcom | Forum website |
| 民心网 | Minxin Website | Forum website |
| 南太湖 | Nan Taihu | Forum website |
| 南太湖论坛 | Nan Taihu Forum | Forum website |
| 南充零距离 | Nanchong zero distance | Forum website |
| 南宫民意通 | Nangong Public Opinion | Forum website |
| 南通热线 | Nantong Hotline | Forum website |
| 新乐山网 | New Leshan Website | Forum website |
| NGA玩家社区 | NGA Gamer Community | Forum website |
| 宁德论坛 | Ningde Forum | Forum website |
| 宁都州人 | Ningduzhouren | Forum website |
| 宁国论坛 | Ningguo Forum | Forum website |
| 宁乡在线 | Ningxiang Online | Forum website |
| 东北新闻网 | Northeast News Network | Forum website |
| 观察者 | Observer Website | Forum website |
| 在线钟祥 | Online Zhongxiang | Forum website |
| 东方财富博客 | Oriental Fortune Blog | Forum website |
| 派代网 | Paidai Website | Forum website |
| 沛县便民网 | Peixianbianming Website | Forum website |
| 百姓问政:榆林人身边事 | People's Question: Yulin People's Side Story | Forum website |
| 平昌零距离 | Pingchang Zero Distance | Forum website |
| 平度论坛 | Pingdu Forum | Forum website |
| 萍乡城事网 | Pingxiangchengshi Website | Forum website |
| 邳州论坛 | Pizhou Forum | Forum website |
| 邳州信息网 | Pizhou Information Network | Forum website |
| 莆田小鱼网 | Putianxiaoyu Website | Forum website |
| 莆仙网 | Puxian Website | Forum website |
| 莆鱼网 | Puyu Website | Forum website |
| 强国论坛 | Qiangguo Forum | Forum website |
| 潜山网 | Qianshan Website | Forum website |
| 汽车之家论坛 | Qichezhijia Forum | Forum website |
| 綦江在线 | Qijiang Online | Forum website |
| 齐鲁民生网 | Qilu Minsheng Website | Forum website |
| 齐鲁网 | Qilu Website | Forum website |
| 秦楚论坛 | Qinchu Forum | Forum website |
| 青海热线 | Qinghai Hotline | Forum website |
| 青青岛社区 | Qingqingdao Community | Forum website |
| 青阳热线 | Qingyang Hotline | Forum website |
| 青阳网 | Qingyang Website | Forum website |
| 戚区网 | Qiqu Website | Forum website |
| 荣耀渭南网 | Rongyaoweinan Website | Forum website |
| 荣耀西安网 | Rongyaoxian Website | Forum website |
| 瑞安在线 | Ruian Online | Forum website |
| 三国源论坛 | Sanguoyuan Forum | Forum website |
| 三明芭乐网 | Sanmingbale Website | Forum website |
| 三明鱼网 | Sanmingfish Website | Forum website |
| 三明kk网 | Sanmingkk Website | Forum website |
| SEO网 | SEO Website | Forum website |
| 柒零叁社区 | Seven Zero Three Community | Forum website |
| 山东001在线 | Shandong001 Online | Forum website |
| 上海论坛 | Shanghai Forum | Forum website |
| 上海证券报·中国证券网 | Shanghai Securities News: China Securities Website | Forum website |
| 山水网论坛 | Shanshui Website Forum | Forum website |
| 汕尾市民网 | Shantou City People's Network | Forum website |
| 汕头e京网 | Shantouejing Website | Forum website |
| 山阳论坛 | Shanyang Forum | Forum website |
| 邵武在线 | Shaowu Online | Forum website |
| 绍兴生活网 | Shaoxing Life | Forum website |
| 绍兴e网 | Shaoxinge Website | Forum website |
| 股民大家庭股票论坛 | Shareholder Family Stock Forum | Forum website |
| 胜利社区 | Shengli Community | Forum website |
| 声远论坛 | Shengyuan Forum | Forum website |
| 嵊州信息港 | Shengzhou Information Port | Forum website |
| 深圳论坛 | Shenzhen Forum | Forum website |
| 深交所互动易 | Shenzhen Stock Exchange Interactive Ease | Forum website |
| 石家庄门户社区 | Shijiazhuang Portal Community | Forum website |
| 时空网 | Shikong Website | Forum website |
| 石台网 | Shitai Website | Forum website |
| 顺德人网 | Shunderen Website | Forum website |
| 四川论坛 | Sichuan Forum | Forum website |
| 泗洪风情网 | Sihongstyle Website | Forum website |
| 新浪爱问 | Sina Ask | Forum website |
| 新浪博客 | Sina Blog | Forum website |
| 新浪股市汇 | Sina Stock Market | Forum website |
| 新浪微博 | Sina Weibo | Forum website |
| 思源社区 | Siyuan Community | Forum website |
| 搜狗问问 | Sogouwenwen Website | Forum website |
| 上证e互动 | SSE eInteractive Website | Forum website |
| 遂宁网 | Suining Website | Forum website |
| 遂平在线 | suiping Online | Forum website |
| 宿松论坛 | Suisong Forum | Forum website |
| 随县论坛 | Suixian Forum | Forum website |
| 随州论坛 | Suizhou Forum | Forum website |
| 松滋100网 | Sungz100 Website | Forum website |
| 阳光博爱论坛 | Sunshine Boai Forum | Forum website |
| 阳光重庆 | Sunshine Chongqing Website | Forum website |
| 宿迁论坛 | Suqian Forum | Forum website |
| 宿迁零距离 | Suqian Zero Distance Website | Forum website |
| 苏州12345阳光便民网站 | Suzhou 12345 Sunshine Convenience Website | Forum website |
| 苏州阳光便民12345 | Suzhou 12345 Website | Forum website |
| 泗阳论坛 | Suzhou12345 Forum | Forum website |
| 宿松百姓论坛 | Suzong People's forum | Forum website |
| 宿松世纪网 | Suzongshiji Website | Forum website |
| 泰安论坛 | Taian Forum | Forum website |
| 大埔网 | TaiPo Website | Forum website |
| 泰有趣 | Taiyouqu Website | Forum website |
| 台州19楼论坛 | Taizhou 19 Floor Forum | Forum website |
| 泰州论坛 | Taizhou Forum | Forum website |
| 泰州问政 | Taizhou Government Q&A | Forum website |
| 畅说108 | Talking 108 Website | Forum website |
| 唐山环渤海新闻网 | Tangshan Bohaihuan News Network | Forum website |
| 陶都论坛 | Tao Du Forum | Forum website |
| 淘股吧 | Taoguba Website | Forum website |
| 百姓之声 | The Voice of the People | Forum website |
| 天目湖论坛 | Tianmu Lake Forum | Forum website |
| 天通苑社区网 | Tiantongyuan Community Network | Forum website |
| 天涯社区 | Tianya Community | Forum website |
| 天涯论坛 | Tianya Forum | Forum website |
| 天一论坛 | Tianyi Forum | Forum website |
| 今日宿州网 | TodaySuzhou Website | Forum website |
| 桐城网 | Tongcheng Website | Forum website |
| 铜梁论坛 | Tongliang Forum | Forum website |
| 潼南论坛 | Tongnan Forum | Forum website |
| 同文译馆翻译之家论坛 | Tongwen Translation House Forum | Forum website |
| 投行先锋 | Touhangxianfeng Website | Forum website |
| 地铁族 | Underground Nation | Forum website |
| 百姓呼声 | Voices of the People | Forum website |
| 玩慈利网 | Wancili Website | Forum website |
| 望江论坛 | Wangjiang Forum | Forum website |
| 望江在线 | Wangjiang Online | Forum website |
| 望京社区 | Wangjing Community | Forum website |
| 网民365 | Wangming365 Website | Forum website |
| 万家热线 | Wanjia Hotline | Forum website |
| 皖江论坛 | Wanjiang Forum | Forum website |
| 微博问答 | Weibo Q&A | Forum website |
| 微博头条 | Weibotoutiao | Forum website |
| 微靖江 | Weijingjiang Website | Forum website |
| 微赞 | Weizan Website | Forum website |
| 魏州网 | Weizhou Website | Forum website |
| 百色看点 | What to see in Baise | Forum website |
| 智慧登封 | Wisdom Dengfeng Website | Forum website |
| 武安之窗 | Wuan Window | Forum website |
| 芜湖民生网 | Wuhumingsheng Website | Forum website |
| 悟空问答 | Wukong Q&A | Forum website |
| 厦门网 | Xiamen Website | Forum website |
| 香山网 | XiangShan Website | Forum website |
| 湘西论坛 | Xiangxi Forum | Forum website |
| 孝感槐荫论坛 | Xiaoganhuaiyin Forum | Forum website |
| 萧内网 | Xiaonei Website | Forum website |
| 小鱼网 | Xiaoyu Website | Forum website |
| 西部网 | Xibu Website | Forum website |
| 西祠胡同 | Xici Hutong | Forum website |
| 新安在线 | Xin'an Online | Forum website |
| 新滨海论坛 | Xinbinhai Forum | Forum website |
| 新昌信息港 | Xinchang Information Port | Forum website |
| 兴宁a8 | Xingninga8 Website | Forum website |
| 行摄玉溪 | Xingsheyuci Website | Forum website |
| 新平之窗 | Xinping Window | Forum website |
| 新三水 | Xinsanshui Website | Forum website |
| 心通桥 | Xintongqiao Website | Forum website |
| 信阳在线 | Xinyang Online | Forum website |
| 信阳网论坛 | Xinyang Website Forum | Forum website |
| 忻州随手拍 | Xinzhousuishoupai Website | Forum website |
| 西蜀论坛 | Xishu Forum | Forum website |
| 西子论坛 | Xizi Forum | Forum website |
| 西子湖畔 | Xizi Lake | Forum website |
| 宣城社区 | Xuancheng Community | Forum website |
| 宣城论坛 | Xuancheng Forum | Forum website |
| 盱眙论坛 | Xuyi Forum | Forum website |
| 盱眙网 | Xuyi Website | Forum website |
| 盐邦论坛 | Yanbang Forum | Forum website |
| 盐城123网 | Yancheng123 Website | Forum website |
| 盐城鹤鸣亭 | Yanchenghemingting Website | Forum website |
| 羊城论坛 | Yangcheng Forum | Forum website |
| 杨梅渡论坛 | Yangmeidu Forum | Forum website |
| 阳泉随手拍 | Yangquansuishoupai Website | Forum website |
| 阳新论坛 | Yangxin Forum | Forum website |
| 扬州生活网 | Yangzhoushenghuo Website | Forum website |
| 盐会网 | Yanhui Website | Forum website |
| 偃师民声网 | Yanshimingsheng Website | Forum website |
| 烟台文化网 | Yantai Culture Website | Forum website |
| 烟台论坛 | Yantai Forum | Forum website |
| 宜宾日报金江网 | Yibin Daily Jinjiang Website | Forum website |
| 宜宾零距离 | Yibin Zero Distance | Forum website |
| 一次办好网上曝光平台 | Yicibanhao: Online Exposure Platform | Forum website |
| 赢家聊吧 | Yingjialiaoba Website | Forum website |
| 易索论坛 | Yisuo Forum | Forum website |
| 亿房网 | Yixiang Website | Forum website |
| 一线生活 | Yixianshenghuo Website | Forum website |
| 宜兴零距离 | Yixing Zero Distance | Forum website |
| 仪征政府论坛 | Yizheng Government Forum | Forum website |
| 乐清城市网论坛 | Yueqing City Net Forum | Forum website |
| 粤西资讯网 | Yuexi Information Website | Forum website |
| 悦西安 | Yuexian Website | Forum website |
| 榆林发布 | Yulin Release | Forum website |
| 运城论坛 | Yuncheng Forum | Forum website |
| 玉溪高古楼 | Yuxi Gao Gu Lou Website | Forum website |
| 余姚论坛 | Yuyao Forum | Forum website |
| 在黄石 | Zaihuangshi Website | Forum website |
| 枣阳论坛 | Zaoyang Forum | Forum website |
| 增城家园 | Zengchengjiayuan Website | Forum website |
| 章丘人论坛 | Zhangqiuren Forum | Forum website |
| 掌上莱州 | Zhangshanglaizhou Website | Forum website |
| 漳州小鱼网 | Zhangzhouxiaoyu Website | Forum website |
| 找法网 | Zhaofa Website | Forum website |
| 镇江论坛 | Zhenjiang Forum | Forum website |
| 镇雄微生活 | Zhenxiongweishenghuo Website | Forum website |
| 浙中在线 | Zhezhong Online | Forum website |
| 知乎专栏 | Zhihu Column | Forum website |
| 知乎 | Zhihu Website | Forum website |
| 中山坦洲人潮网 | Zhong Shan Tan Zhou Ren Chao Website | Forum website |
| 中吴网 | Zhongwu Website | Forum website |
| 钟祥论坛 | Zhongxiang Forum | Forum website |
| 周口论坛 | Zhoukou Forum | Forum website |
| 诸暨在线 | Zhuji Online | Forum website |
| 驻马店广视网 | Zhumadian Guangshi Website | Forum website |
| 株洲新闻网 | Zhuzhou News Website | Forum website |
| 株洲在线 | Zhuzhou Online | Forum website |
| 株洲草根网 | zhuzhougrassroots Website | Forum website |
| 自贡在线 | Zigong Online | Forum website |
| 最耒阳论坛 | Zuileiyang Forum | Forum website |
